# Active Learning Improves Ionization Efficiency Predictions and Quantification in Nontargeted LC/HRMS

**DOI:** 10.1021/acs.analchem.5c00816

**Published:** 2025-06-13

**Authors:** Wei-Chieh Wang, Nahid Amini, Carolin Huber, Meelis Kull, Anneli Kruve

**Affiliations:** † Department of Chemistry, 7675 Stockholm University, Svante Arrhenius väg 16, 114 18 Stockholm, Sweden; ‡ Oriflame Cosmetics, Scientific Research & Technology, Fleminggatan 14, 112 26 Stockholm, Sweden; § Department of Exposure Science, 28342 Helmholtz Centre for Environmental Research − UFZ, Permoserstr. 15, 04318 Leipzig, Germany; ∥ Institute of Computer Science, 37546 University of Tartu, Narva mnt 18, 51009 Tartu, Estonia; ⊥ Department of Environmental Science, Stockholm University, Svante Arrhenius väg 8, 114 18 Stockholm, Sweden

## Abstract

Liquid chromatography electrospray ionization high-resolution mass spectrometry (LC/ESI/HRMS) is frequently employed in nontargeted screening (NTS) due to its high selectivity and sensitivity. However, data interpretation is challenging since the number of chemical standards available for quantification is limited and the response of the chemicals vastly differs depending on their structure and analysis conditions. Therefore, machine learning (ML) models have been utilized to predict ionization efficiency (*IE*) and enable the quantification of detected chemicals. It has been observed that the error in the predictions is high for chemicals structurally different from the training data. To enlarge the training set and to accurately predict the IE given a limited labeling budget, active learning (AL) is proposed to acquire informative data points from the targeted chemical space. In the current study, four AL approaches (clustering-based, uncertainty-based, mix, and anticlustering) and a baseline approach (random) were evaluated for *IE* prediction. The RMSE of the *IE* in the targeted space dropped significantly (up to 0.3 log units) after a single AL iteration, highlighting the necessity of chemical space exploration before ML model execution. Clustering-based AL reduced the RMSE least, while the uncertainty-based AL was inefficient if ten or more chemicals were sampled in one iteration, thereby reducing its practicality. Finally, expanding the chemical space improved the quantification accuracy from a fold error of 4.13× to 2.94× for five natural products in *Alpinia officinarum*, thereby demonstrating the need for updating the chemical space coverage of the training set.

## Graphical abstract

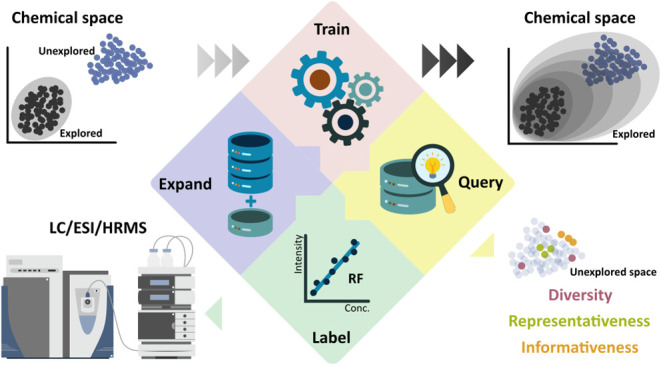

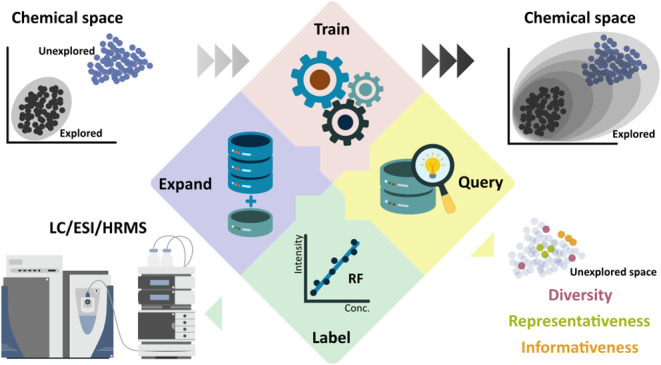

## Introduction

Discovering emerging chemical contaminants in the environment, active natural products, or metabolites associated with biological responses is increasingly relying on nontargeted screening (NTS) with liquid chromatography high-resolution electrospray ionization mass spectrometry (LC/ESI/HRMS).^1–3^ In such samples, NTS routinely enables the detection of thousands of features; however, confirmation and quantification with analytical standards are often possible for only up to 10% of detected features.^4,5^ Machine learning (ML) models have been developed to overcome this obstacle and enable prioritization of potentially hazardous chemicals detected with NTS in the samples.^6^ For instance, the toxicity^7–9^ and concentration^10,11^ can be predicted to pinpoint the most potentially hazardous chemicals instead of selecting the chemicals based solely on the signal intensity.

The fundamental assumption of ML is that the data used for the model training are representative and informative of the predicted property;^12^ therefore, the applicability of ML models depends on the chemical space of the training data. Concerningly, the chemical space expands from metabolomics to environmental monitoring but also across sample types within the same research field (water vs soil, urine vs blood).^13^ A recent analysis by Kretschmer et al.^14^ evaluated, based on the maximum common edge subgraph distance, that the majority of data sets used for machine learning model training in LC/HRMS show only a partial overlap with the often-targeted chemical space of biomolecules.

Simultaneously, leveraging already trained models may be advantageous as general associations between descriptors and response are likely to apply across chemical classes, i.e., polarity is the main driver in retention time (RT) predictions in reversed-phase LC for pharmaceuticals,^15^ and per- and polyfluorinated chemicals^16^ (PFAS) alike. The former study trained multilinear regression and support vector machine models on 168 data points to predict RT for eight aminopyridines, and the latter one used 58 observations from measurements of PFAS to predict RT. Nevertheless, ML models need to be applicable in the specific application domains. For example, we have recently shown that the chemical space of ionization efficiency (*IE*) prediction models can be effectively enlarged toward hydroxylated polychlorinated biphenyls by adding just a few representative chemicals to the training set while training a new model only based on targeted chemical space would require tens or hundreds of measurements that might remain unreachable due to a limited set of analytical standards.^17^ However, what is left unclear is which chemicals and how many need to be measured to increase the prediction accuracy most effectively.

In response to the need to understand and expand the application domain of ML models, the evaluation of the properties of training data, such as representativeness, diversity, and informativeness, is increasingly acknowledged. Noticeably, representativeness is primarily studied for image recognition. For example, Schat et al.^18^ evaluated the similarity of brain tissue segmentation in magnetic resonance imaging data and the new unseen data. They proposed the *Data Representativeness Criterion index* to assess the performance of the model and indicate the insufficiency of the training data. Informativeness is a relatively subjective property defined as the data points that provide the most novel information, and such points may differ from one ML model to another. As a result, Bressan et al.^19^ measured informativeness as the uncertainty associated with a specific data point in a particular model, where higher uncertainty indicates higher informativeness. Additionally, diverse data sets have been suggested to offer more information for ML models.^20^ Glavatskikh et al.^21^ compared the generalizability of the ML models trained on data sets with different degrees of diversity. The model trained on more diverse chemicals generated predictions for chemicals in the other data set with lower mean absolute errors on the chemical energies, such as nuclear repulsion energy.

Active learning (AL) is an approach that iteratively chooses new training instances to improve the performance of the model.^22,23^ First, it pinpoints the chemicals that most effectively expand the chemical space of training data. Thereafter, the new data are experimentally labeled and added to the original training data, followed by retraining the model. This workflow could be implemented iteratively until the cost limit (time, money) is reached or the desired model performance is achieved. AL has been widely applied to improve model performance in image recognition and lead discovery in drug design.^24,25^ From these studies, AL has shown the capability of boosting the performance of ML models and the potential of accelerating the process of chemical space exploration. Though various AL approaches and data sets have been examined, the primary purpose of these studies has been to navigate the ML models toward identifying the most promising candidates in drug discovery or material design. This research question fundamentally differs from the need to improve prediction accuracy in the whole predicted property range, e.g., in NTS, the accurate prediction of both high and low *IE* chemicals is required for accurate quantification and reliable prioritization.

In the present study, we aim to (1) expand the coverage of the chemical space for *IE* prediction, (2) reduce the prediction errors for the targeted chemical space, and (3) evaluate the performance of different AL approaches in simultaneously achieving (1) and (2). Sampling approaches, such as random sampling, uncertainty-based sampling,^26^ clustering-based sampling, anticlustering sampling,^27^ and a mixed algorithm, which combines clustering- and uncertainty-based sampling, were compared for a different number of chemicals sampled in each iteration (sampling number, *n*_sample_), from 1 to 20. Furthermore, we investigate the impact of AL approaches on the descriptors used by the prediction model. Last but not least, we evaluate the impact of training set chemical space on the quantification in NTS by quantifying ten natural products in *Alpinia officinarum* with *IE* prediction models before and after AL.

## Method

### Data Preparation

The PaDEL descriptors, designed by Yap,^28^ were used to numerically depict the chemicals. All data sets were cleaned and scaled to reduce the number of chemical descriptors with low or redundant information. The features containing information about the eluent composition, such as pH, the presence of ammonium, polarity, viscosity, and surface tension, were excluded from the cleaning process. Descriptors with near-zero variance (cutoff 80/20) or high correlation (0.75) were removed with *nearZeroVar* and *findCorrelation* functions from the *caret* (version: 6.0-94) package in R Studio (version 2024.04.0 + 735), respectively.^29^ All descriptors were scaled after each AL iteration for the training set, and the same scaling was applied to the unexplored chemical space (for short, “unexplored space”). The same operation was applied for the uniform manifold approximation and projection (UMAP) and distance computation.

The similarity of the unexplored space to the explored chemical space (for short, “explored space”) was calculated as the Euclidean distance from each of the chemicals in the unexplored space to the five closest chemicals in the explored space based on scaled and entered PaDEL descriptors. Additionally, the same five nearest neighbors distance (*d*_NN=5_) was calculated inside the explored space.

The log *IE* prediction model training process was adapted from previous publications; therefore, extreme gradient boosting (xgBoost) was used to train the log IE prediction models.^11^ ,^30^ ,^31^ The model was trained on log *IE* values measured in the negative ESI model. The original training set contained 81 unique chemicals measured with different mobile phase compositions, e.g., pH values, buffer, and organic modifier content, yielding 1009 instances for the explored space. These chemicals, mainly carboxylic acids and phenols, were considered as the explored space. The unexplored space contained four data sets: PFAS, hydroxylated polychlorinated biphenyls (OH-PCBs), natural products, and environmental contaminants (pesticides, pharmaceuticals, and ingredients of personal care products, etc.). The former two data sets have been previously published by Lauria et al.^30^ and Khabazbashi et al.,^17^ respectively. The latter two were measured here, and a detailed description of data acquisition is given in the Supporting Information. Altogether, these data sets, forming the unexplored space, contained 369 chemicals. Furthermore, the models trained on all available new data are compared on ten independently analyzed chemicals (Table 1), forming a completely independent validation of the newly trained models.

**Table 1:** Experimental and Predicted log IE Values and Concentrations before and after Chemical Space Exploration for the 10 Targeted Chemicals (Five with Reference Standards) in *Alpinia officinarum* Extract

		before AL		after AL	
chemicals	*C*_ref_ (μM)	log *IE*_pred_	*C*_pred_ (μM)	log *IE*_pred_	*C*_pred_ (μM)
pinocembrin	15	0.28	34	–0.99	37
(−)-epicatechin	23	–0.60	78	–1.55	41
kaempferol		1.04	1.2	–0.29	2.4
galangin	110	1.25	66	0.06	146
luteolin-3′,7-diglucoside	0.16	–0.51	13	–0.36	6
catechin	1.2	0.49	1.1	–0.07	1.2
pinobanksin		0.63	33	–0.46	45
kaempferide		1.10	24	–0.56	51
galangin 7-glucoside		0.83	7.8	0.10	11
galangin 3-rhamnoside		–0.06	2.2	–0.23	1.6

The prices of the chemicals were obtained from Merck (https://www.sigmaaldrich.com/SE/en) between the third and sixth of November 2024. The price of the product with the purity of analytical standards or above was recorded and used for the evaluation of chemical accessibility. The in-house synthesized chemicals, mostly in the OH-PCBs data set, were considered inaccessible and not considered. The final combined data set and the source codes are available at https://github.com/kruvelab/MS2Quant.

### Sampling Method

*Random sampling*, a baseline approach, alongside four AL approaches, was evaluated. These AL approaches suggested chemicals based on the diversity, representativeness, and informativeness of the data set.^32^ First, sampling based on the *k*-means clustering algorithm was utilized. Here, the number of clusters equaled the number of chemicals to be sampled per iteration, and the chemicals closest to the cluster centers were sampled as the representative chemicals for each cluster. Second, the uncertainty-based AL leveraged a *quantile regression forest algorithm* to compute the log *IE* prediction intervals for each chemical in the unexplored space.^26^ The chemical with the largest prediction interval was considered the most informative and was sampled. Third, *an anticlustering sampling algorithm* was utilized to sample chemicals with the highest diversity.^27^ While the *k*-means clustering was implemented for obtaining the most representative compounds, the anticlustering algorithm generates clusters by simultaneously maximizing the internal distance and minimizing the external distance, and generates selections based on diversity.^27^ In each iteration, all chemicals in one random cluster were sampled. Fourth, a *mix sampling approach* was designed. This approach aimed to balance informativeness and representativeness; therefore, the unexplored space data set was first clustered based on the desired *n*_sample_. The mean distance to the cluster center was calculated for each data point in each cluster. These mean distances were then subjected to a second *k*-means clustering (*k* = 2), where one group was classified as sparse and the other as dense. The *uncertainty-based* and *clustering-based sampling approaches* were employed for the sparse and dense clusters, respectively. The sampling approaches were repeated in each iteration, as described above. Additionally, a cost-based sampling approach was evaluated in the case *n*_sample_ = 20 with five iterations and 50 repetitions to mimic the chemical availability in a real case in the chemical labeling step. The top 20 cheapest chemicals were selected in each iteration, and the averaged RMSE_pooled_ of the test set was compared.

### Performance Evaluation

The performance of the models trained with each AL approach was evaluated on a targeted chemical space (for short, targeted space), which was simulated by a randomly sampled test set from the unexplored space (80/20) prior to AL. Thereafter, the data set of the explored space was cleaned and scaled to avoid data leakage, and the same scaling was applied to the unexplored space and the test set (targeted space). The cleaning was conducted each time the data sets were updated by AL to adapt to the larger training set. To reduce the impact of the random selection of chemicals in the test set, the sampling and the following active learning iterations were repeated 50 times. In each repetition, 20 active learning iterations per *n*_sample_ were performed. Meanwhile, a space-separated cross-validation was designed to evaluate the influence of different explored-unexplored space pairs on the performance of the AL. The detailed method is summarized in the Supporting Information. Furthermore, the models trained on all available new data are compared on ten independently analyzed chemicals (Table 1), forming a completely independent validation of the newly trained models.

Due to the extensive chemical differences and the unequal number of chemicals in the four data sets, the pooled root mean squared error (RMSE_pooled_) (eqs 1 and 2) was used to evaluate the performance of the trained models. In brief, the RMSE was first calculated separately for chemicals in the test set from the PFAS, PCB, natural product, and environmental contaminants data sets and subsequently pooled. Furthermore, the standard error of the mean (SEM) was used to examine the stability of the performance across 50 repetitions (eqs 3 and 4).

$$\mathrm{RMSE}=\sqrt{\frac{\overset{n}{\underset{1}{\sum }}{\left({\log IE}_{\mathrm{predicted}}-{\log IE}_{\mathrm{experimental}}\right)}^{2}}{n}}$$ 1

where *n* equals the number of chemicals in a specific chemical class

2 $${\mathrm{RMSE}}_{\mathrm{pooled}}=\sqrt{\frac{{\sum_{1}^{m}\mathrm{RMSE}}_{i}^{2}}{m}}$$

where *m* refers to the number of classes in the unexplored space

$$s=\sqrt{\frac{\sum {\left({\mathrm{RMSE}}_{\mathrm{pooled}}\;-{\overset{®}{\mathrm{RMSE}}}_{\mathrm{pooled}}\;\right)}^{2}}{N}}$$ 3

4 $$\mathrm{SEM}=\frac{s}{\sqrt{N}}$$

where *N* means the number of repetitions.

## Result

### Similarity between the Explored and the Unexplored Spaces

The similarity of the explored and the unexplored spaces was quantitatively evaluated based on the distribution of the distance between the two spaces as well as visually with UMAP.^33^ The visualization of the chemical spaces with UMAP (Figure 1A) indicated poor overlap between the explored and unexplored spaces and implied the necessity of chemical space exploration. In the unexplored space, the well-separated small cluster at the middle bottom of the UMAP representation contained 32 chemicals, incorporating PFAS from the designated PFAS data set and environmental contaminant data set (e.g., heptafluorobutanoic acid).

**Figure 1:**
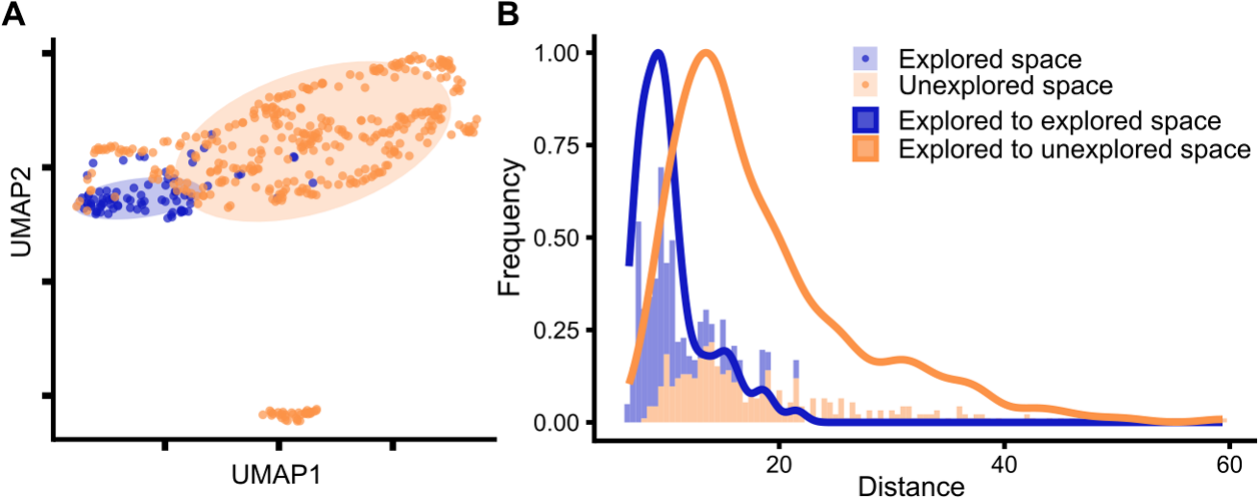
(A) The explored and unexplored spaces in UMAP embedding. The colored ellipses indicate the 68% confidence region of the data set. (B) Distance distribution from the explored to the explored space and from the explored to the unexplored space.

Additionally, the degree of overlap between the explored and the unexplored spaces was quantitatively evaluated by *d*_NN=5_ (Figure 1B). Confirming the conclusions from UMAP, the distances from the explored space to itself lay within 6.67 to 21.50 units, while the distances from the unexplored space to the explored space ranged from 8.09 to 59.36 units. The 95% quantiles of the distances were 16.72 and 36.17 units, respectively.

### Evaluation of Sampling Approach Algorithms

For uncertainty-based AL, the interval of the predicted log *IE* was used as an approximation of the uncertainty, where a larger interval indicates higher uncertainty. It was observed that the prediction interval increased with the distance from the explored space and leveled off after a *d*_NN_ of 20 (Figure S1).

The *k*-means clustering yielded clusters of chemicals closely aligned with their chemical classes derived from ClassyFire^34^ (Figure S2). Evidently, none of the chemical classes was distributed across all clusters, and many clusters had one dominant chemical class, with more than 50% of chemicals belonging to that class. It is acknowledged that using the same sampling approach could result in sampling a redundant chemical space, which means sampling similar chemicals across iterations.^35^ The mix sampling combines clustering- and uncertainty-based AL. In the first iteration and the first repetition with *n*_sample_ = 5, four clusters were classified as dense clusters, and one as a sparse cluster (Figure S3). Accordingly, chemicals with the largest prediction interval in the sparse clustering were selected for labeling.

### Comparison of the Performance of the Sampling Approaches

The maximum number of the selected chemicals with 20 iterations were 20, 100, 200, 296, and 296 for sampling one, five, ten, 15, and 20 chemicals per iteration, respectively, leading to 6.79, 33.78, 67.57, 100, and 100% of the chemicals sampled from the unexplored space. The initial RMSE_pooled_ before AL was, on average, 1.68 log *IE* units and dropped significantly after the first AL iteration, regardless of the sampling approach or number of chemicals sampled (Figure 2A). The mean RMSE_pooled_ decreased to 1.22–1.25 log *IE* units after sampling approximately 6% of the chemicals, and reached down to 0.99 log IE unit depending on the algorithms for other *n*_sample_ after 20 iterations.

**Figure 2:**
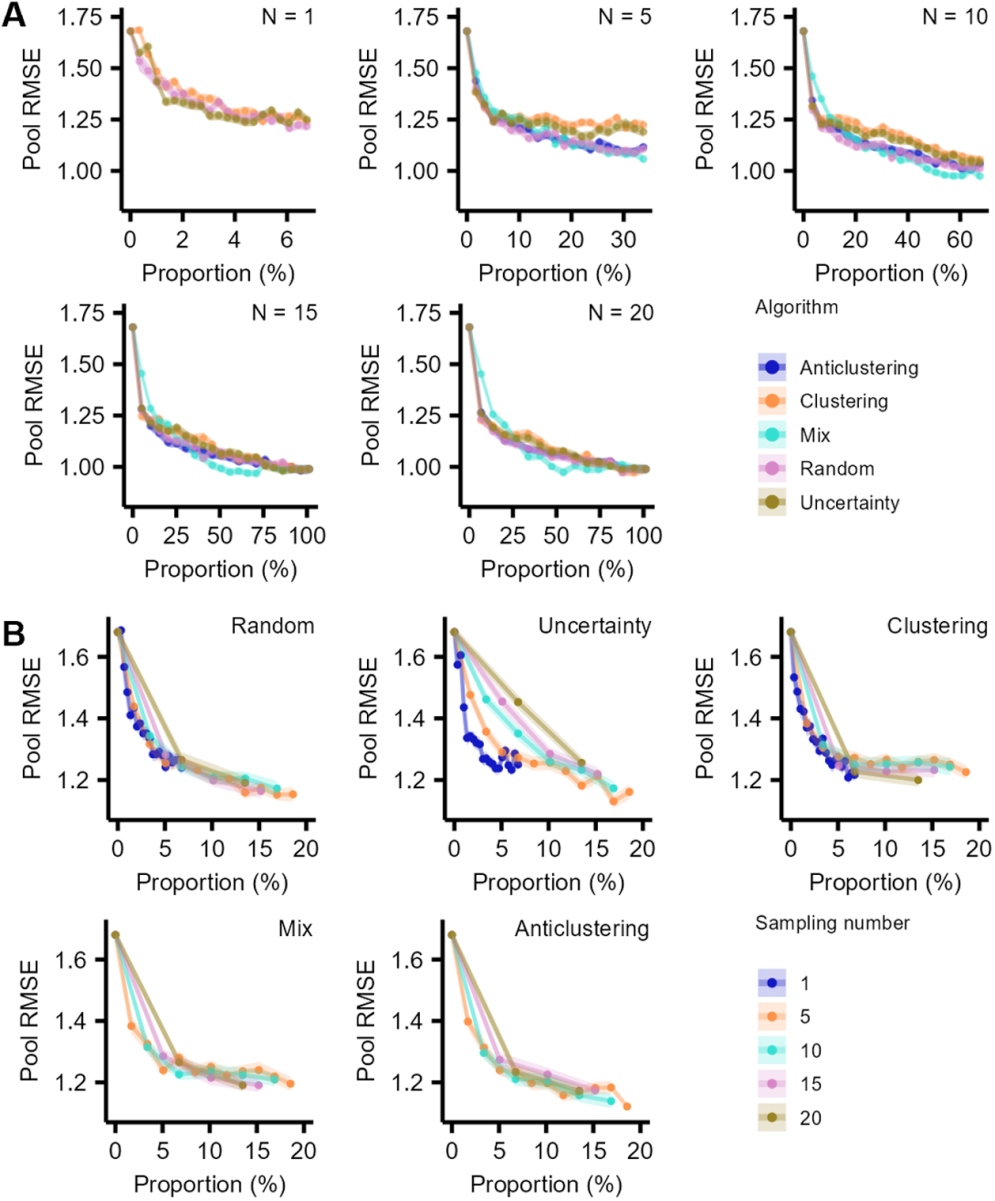
(A) AL performance evaluation using selected algorithms and various *n*_sample_. (B) AL performance evaluation using selected *n*_sample_ and different algorithms. The *x*-axis represents the proportion of labeled chemicals over the total chemicals in the unexplored space at each iteration step. As the final prediction performance was similar, the proportion in (B) was limited to 20% to showcase the influence of different numbers in the early stages of AL. Each point in the plots represents the average result for one iteration across all repetitions, and the color ranges were inferred from the SEM. The upper and lower values of the SD of the RMSE_pooled_ were determined by SEM.

The trend of decreasing test set RMSE_pooled_ with each AL iteration was supported by the increasing *R* ^2^ (Figures S6–S9). The *R* ^2^ between the experimental and the predicted log *IE* was 0.14 without AL and increased to 0.59–0.61 for all AL approaches when all chemicals were labeled in the AL workflow (Figure S9). No significant differences in the improvement in RMSE_pooled_ and *R* ^2^ were observed among different chemical classes (Figures S6–S9). Nevertheless, differences in the performance of AL approaches were observed depending on the number of iterations and the number of labeled chemicals per iteration.

It was observed that anticlustering and random sampling improved prediction accuracy more than clustering-based and mix sampling. Clustering-based and mix sampling approaches showed no clear improvement in RMSE_pooled_ when labeling 10–35% chemicals. The RMSE_pooled_ remained higher for clustering-based and mix sampling in comparison to anticlustering and random sampling with *n*_sample_ = 10 for labeling 10–70% of the chemicals; however, for *n*_sample_ = 15 and *n*_sample_ = 20, no significant differences were observed between these approaches. Lastly, the uncertainty-based sampling yielded the least improvement in RMSE_pooled_ for the first few iterations. Simultaneously, clearly lower RMSE_pooled_ values were obtained for 30–75% of labeled chemicals in the targeted space. This observation implied that the approaches were capable of capturing more information from the unexplored space compared to the other approaches.

Furthermore, the *n*_sample_ value substantially affected the performance of the AL (Figure 2B). The RMSE_pooled_ decreased substantially after the first iteration, regardless of the number of selected chemicals; however, the least improvement was observed with uncertainty-based AL. This indicates that a smaller *n*_sample_ is more suitable for uncertainty-based AL. It was hypothesized that this outcome resulted from the insufficient diversity of the labeled chemicals when using a larger *n*_sample_, which was further investigated for all AL approaches.

Results from the space-separated cross-validation implied a similar pattern that the RMSE_pooled_ generally dropped after the first AL iteration, especially if the natural product and the PCBs and PFAS data set were considered as the explored space. The results confirmed the observation that AL could improve the performance of the ML models, and it would be important to conduct chemical space exploration prior to generating property predictions (Figure S4). Additionally, a larger standard error of RMSE was observed in the case when the size of the explored space, such as the PCB and PFAS data sets, was smaller. On contract, the explored space with more instances, such as the non-PFAS data set, had the narrowest width ribbon areas across all AL strategies, even though there were fewer unique compounds in the non-PFAS data set compared to others.

Similar to the results in the original AL estimation, uncertainty-based sampling behaved similarly in the first iteration in the space-separated cross-validation (Figure S5). The RMSE_pooled_ reached the close level after the first iteration with various *n*_sample_, indicating the deficiency of compound diversity in the uncertainty-based sampling with larger *n*_sample_.

### Evaluation of the Chemical Space Exploration Efficiency

It was hypothesized that the different performance of the AL approaches may stem from chemical space exploration. Clustering and uncertainty-based approaches showed substantially different behaviors in chemical space exploration compared to random sampling (Figure 3). The clustering-based approach, which aims to sample the most representative chemicals, labeled the chemicals at the center of the densest area for *n*_sample_ = 1 (Figure 3). In the following iterations, even though the center of the cluster would slightly shift due to the decreasing number of unlabeled chemicals, the sampled chemicals remained close to those already selected. This clearly led to sampling within a narrow chemical space (ellipse of the labeled chemicals in Figure 3). Clustering-based AL sampled a diverse chemical space in each iteration if *n*_sample_ > 1. Nevertheless, similar to *n*_sample_ = 1, the sampled data points were close to those investigated in the previous iterations (Figures S11–S14).

**Figure 3:**
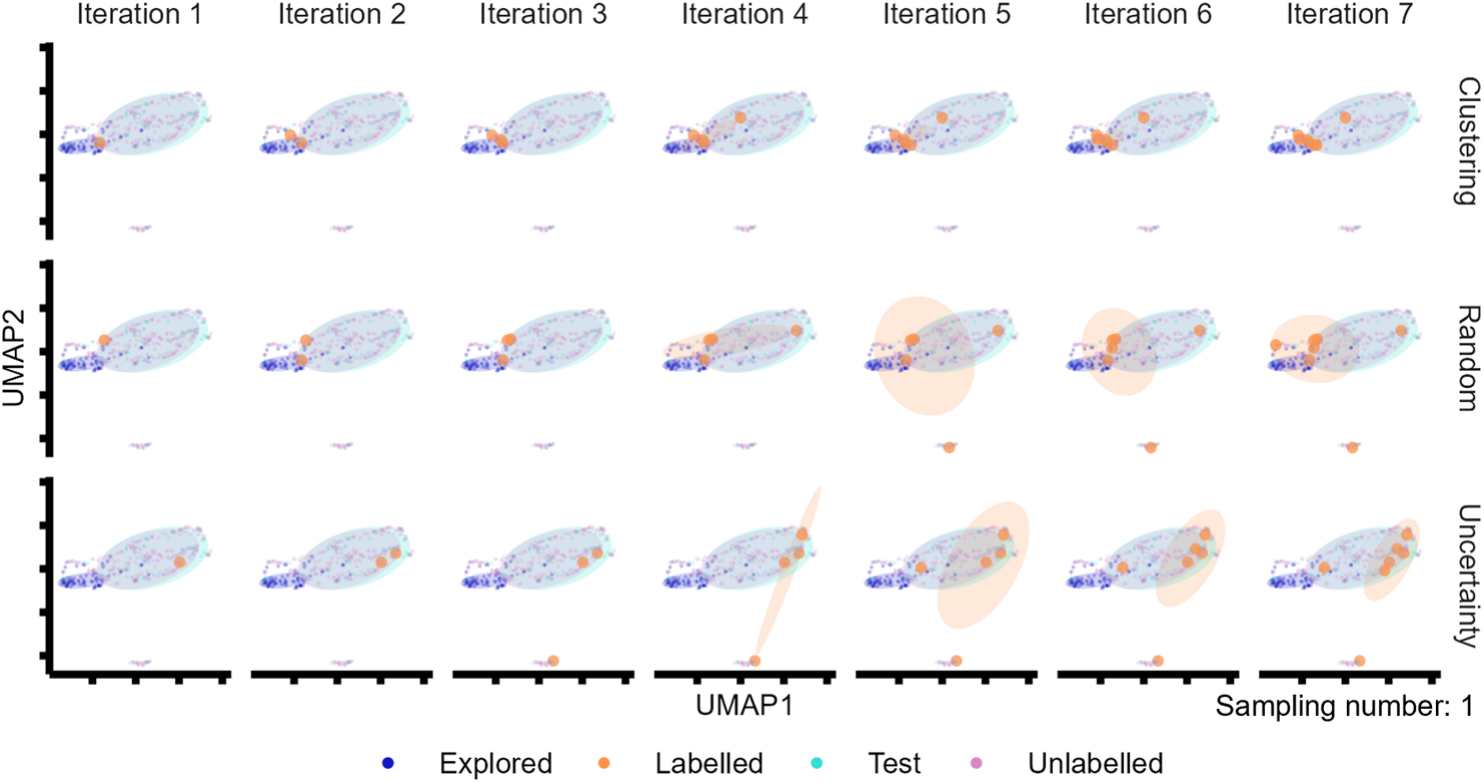
Chemical space exploration in the early stages of AL (from the first to the seventh iteration) with *n*_sample_ = 1. One additional chemical was labeled in each iteration in the plot. The first repetition was selected as the representative for the evaluation. Parameters for ellipse computation: t-distribution with a level equal to 0.68. Highly overlapping areas between the testing points and the unlabeled points indicate a good representativeness of the targeted space (test set).

On the other hand, the uncertainty-based AL sampled the chemicals furthest away from the explored space (Figures 3 and S11–S14). While the clustering-based AL lacked diversity in sampled chemicals from iteration to iteration, the uncertainty-based AL approach lacked diversity in the chemicals sampled within one iteration. Therefore, the uncertainty-based AL has low efficiency in chemical space exploration for a low number of iterations, while the performance of clustering-based AL levels off after a few iterations. Noticeably, the uncertainty-based AL was the only approach that suggested the chemicals from the PFAS area (a cluster of points on the lower middle of UMAP in Figure 3) in this specific repetition in the first few iterations.

The mix and anticlustering approach could not be executed with *n*_sample_ = 1, so *n*_sample_ = 5 was investigated instead (Figures S11–S14). Similar to clustering-based AL, these approaches sampled chemicals located in different areas of the unexplored space as they leverage clusters generated prior to the sampling (Figure S11).

### Evaluation of the Variable Importance

The AL aims to provide more information for ML model training and thereby impact the descriptors used by the models. The baseline model, trained prior to AL, used 117 descriptors (Figures S15–S37). During AL, the training data was cleaned separately in each iteration, impacting which and how many descriptors were available to the model training. Generally, new descriptors were continuously being introduced to the models, with the exception of the clustering-based sampling (Figure S25). The number of descriptors reached its maximum after sampling ∼200 (∼67.57%) chemicals, whereas models trained after random sampling incorporated more descriptors than other sampling approaches (Figure S38).

In the example of *n*_sample_ = 15, the variable importance of 34 of the initially incorporated descriptors decreased to zero, while 138 new descriptors were added, leading to a total of 255 descriptors with clustering-based sampling (Figure S28). Furthermore, the ranking of the descriptors based on variable importance changed. For example, the descriptor “ASP.6” (d1064 in Figure S28) was the most important variable in the original models, while the descriptor “AATS5m” (d1021) yielded the highest mean variable importance after the first iteration. The majority of the top 20 descriptors that were important in the initial model showed low variable importance after the introduction of new chemicals. For instance, the descriptor BCUTw.1l (d25) became less influential in the later iterations.

Some descriptors were introduced later to the models. Descriptor ZMIC5 (d457), which was removed during data cleaning in the initially explored space data set, yielded a nonzero mean variable importance after the fifth iteration and became one of the highest-ranked variables in the second half of AL. Descriptor AATS2s (d1183) was introduced at the end of the first half of AL; however, its average variable importance decreased back to zero after six iterations. From the perspective of the types of descriptors, descriptors belonging to “autocorrelation” were introduced earlier, and the descriptors from “atom type electrotopological state” were introduced later.

Interestingly, the mobile phase descriptors (pH, polarity, surface tension, viscosity, and the presence of NH_4_^+^, denoted d1 to d5) ranked below the 10th position, with the exception of pH, which was fourth based on variable importance. Among these five parameters, the presence of NH_4_^+^ is a discrete descriptor and yielded the lowest variable importance.

### Quantification Accuracy before and after Chemical Space Exploration

The aim of log *IE* prediction is to facilitate the quantitative analyses if analytical standards are unavailable; therefore, ten natural products in *Alpinia officinarum* extract were quantified using the log *IE* models before and after AL. All chemicals with available log *IE* values were used to train the model after including all new chemicals, except the five chemicals present in the sample. The predicted log *IE* values were lower afterward for most chemicals, except luteolin-3′,7-diglucoside (Table 1). The fold errors of the concentration prediction ranged from 1.13× to 81× before active learning and decreased with active learning, ranging from 1.01× to 37×. The largest improvement in quantification accuracy was observed for luteolin-3′,7-diglucoside. In both cases, the majority of the predicted concentrations were overestimated. Though accurate predictions are always desired, overestimations can be more tolerated in some applications, such as risk assessment, and less tolerated in others, such as process engineering for active chemical extraction.^36^

## Discussion

Five sampling approaches were evaluated in the study for their efficacy in chemical space exploration based on the improvement in the prediction error. Furthermore, coverage of the targeted space and the descriptors used by the trained models were evaluated based on variable importance. The effect of training data size was assessed by varying the *n*_sample_, which also allowed us to consider the time and cost required for labeling. The importance of chemical space exploration for ML models was demonstrated by applying the *IE* prediction ML models to the analysis of *Alpinia officinarum* extract before and after the chemical space exploration.

### Performance Evaluation of the AL

The AL approaches suggested chemicals based on diversity, informativeness, and representativeness. The first was approached through anticlustering, while the latter two were central to uncertainty-based and clustering-based AL, respectively. To ensure the diversity of the data points sampled in each iteration of clustering-based sampling, the targeted space was first divided into several subsets based on Euclidean distance. Nevertheless, in subsequent iterations, both clustering- and uncertainty-based approaches sampled adjacent chemicals, leading to a narrow chemical space coverage.

Sampling more chemicals simultaneously in LC/HRMS analyses is time- and resource-efficient, suggesting that sampling more chemicals per iteration is advisable, provided that the model performance remains unaffected. In the case of uncertainty-based AL, the RMSE_pooled_ did not substantially improve with a higher *n*_sample_ for the same iteration number (Figure 2B), which could be associated with the diversity of the chemicals sampled by the AL approach. The chemicals located furthest from the explored space yielded the highest uncertainty with the original model and were therefore considered the most informative; however, all these chemicals were located closely in chemical space, leading to a lack of diversity. In other words, the chemical information available to the models was similar across sampling numbers in the first iterations. Therefore, uncertainty-based AL became inefficient if the number of iterations was limited.

Clustering-based AL (*n*_sample_ > 1) sampled chemicals from different areas of the chemical space, leading to efficient sampling in the first iterations. However, it continued to sample similar chemicals in the subsequent iterations (Figures S11–S14). This phenomenon resulted in minimal or no further improvement in RMSE_pooled_ after sampling ∼ 10% of chemicals of the unexplored space, while other approaches yielded a continuous decrease in RMSE_pooled_ (Figure 2B). The RMSE_pooled_ began decreasing again after sampling over 50% of chemicals from the unexplored space with clustering-based AL. The impact of sampling redundant chemical space was also evident from the number of descriptors used by the ML model for each AL approach. The final numbers of descriptors used by the models generated from clustering-based, uncertainty-based, and random sampling approaches after 20 iterations (*n*_sample_ = 1) were, on average, 123, 158, and 161, respectively (Figure S38). Even more so, only six new descriptors were introduced to the model with clustering-based AL. Overall, the redundant chemical space sampling associated with clustering-based AL sampling should be carefully considered when utilizing the approaches and cannot be recommended for intermediate iteration numbers.

Mix sampling was designed to balance the advantages of clustering-based and uncertainty-based sampling approaches. Despite the combination, mix sampling also sampled chemicals from a narrow chemical space and yielded only a moderate decrease in RMSE_pooled_ in later iterations. This outcome indicated that mix sampling did not fully capitalize on the benefits of uncertainty-based sampling in later stages of AL.

The anticlustering approach, which shares the same advantage as the clustering-based approach when n_sample_ > 1, ensured the diversity of chemicals sampled within each iteration. However, the diversity of sampled chemicals between iterations was low. In our experiment, we randomly assigned one of the anticlusters for labeling. It could be recognized that the chemicals labeled in the next iterations were close to those labeled in previous iterations (Figures S11–S14). Chemicals sampled by the anticlustering approach failed to increase diversity in the explored space (Figure 2A), contributing to the observation that uncertainty-based AL outcompeted anticlustering when 40–75% of the chemicals in the unexplored space were sampled. In summary, sampling redundant chemical space is a recurring problem in AL for chemical space exploration.

Generally, random and anticlustering approaches showed lower RMSE_pooled_ values and a greater capacity to explore the chemical space, emphasizing the importance of diversity of the training data in ML procedures. A similar trend was observed previously by Fine et al.,^37^ in which a model trained with a relatively small but structurally diverse training set yielded better performance than models trained with structurally similar training sets. In the current study, the low RMSE_pooled_ values observed with random sampling could be attributed to the relatively small unexplored chemical space. In a smaller chemical space, the covered chemical space is proportionally larger compared to a larger space under the same set of chemicals. However, the chemicals selected by random sampling covered a relatively smaller chemical space than those from anticlustering after the first iteration (Figures S11–S14). Although the subspace was enlarged in the following iterations, random selection may suggest chemicals within a narrow chemical space (Figure S40). Namely, since the unexplored space was unevenly distributed, the likelihood of random selection sampling chemicals from sparse regions was lower, indicating a potential drawback of random sampling.

In practice, it may be tempting to expand the chemical space by labeling the most available chemicals, e.g., based on cost. Therefore, an assessment for cost-based sampling was conducted (Figure S41). Cost-based sampling surpassed only uncertainty-based sampling in the first iteration and was associated with insufficient diversity of sampling chemicals with the cost-based approach. Subsequent iterations of cost-based sampling marginally improved the RMSE_pooled_ and performed significantly worse than the other approaches. This indicates that the most accessible (low cost) chemicals are likely more similar to one another and introduce little novel information to the model. Additionally, the increased RMSE_pooled_ in the fifth iteration could imply overfitting and inefficient chemical space exploration.

Despite the improvement in model performance through chemical space exploration, it was observed that the applicable domains of the models could be affected when novel information was added to the original training set during the AL workflow (Figure S42). Namely, the RMSE_pooled_ increased after the first iteration for a test set sampled from the explored space. We hypothesize that this suggests a substantial difference in the ionization mechanism or measurement procedure used to obtain log *IE* values for the targeted and explore chemical space. Previous research has shown that ESI source geometry can significantly impact the log *IE* values.^38^ This phenomenon underscores the need to balance the training data from both the explored and unexplored chemical spaces if a general model is desired, e.g., in the case of NTS, where models are utilized to predict log *IE* for chemicals spanning a wide range of chemical property values.

### Variable Importance Analysis

Regarding the descriptors used by the models, some became insignificant while others were introduced as the number of data points in the training set increased, regardless of the AL approach. It was observed that new descriptors were introduced at similar iterations across different AL approaches and exhibited comparable trends. For example, the descriptor WPATH (d555), which belonged to the Wiener numbers category, was consistently included during the early stages of AL and became less important in the later stages. Meanwhile, descriptor ZMIC5 (d457) from the information content panel was typically added in iterations six to seven and became increasingly important in subsequent iterations. Unlike other relatively important descriptors, descriptor AATS 2s (d1138) was only used by the ML model between the seventh and 15th iterations of AL, regardless of the sampling approach or *n*_sample_. The significant differences in the descriptor values distribution between the explored and the unexplored space could account for this gradual introduction of the descriptors (Figure S43). Among these three descriptors, WPATH (d555), which was introduced first, possessed the most pronounced difference. In contrast, the descriptors ZMIC5 (d457) and AATS 2s (d1138) had more similar distributions across the two chemical spaces.

The variable importance changes of the descriptors manifested the model’s gradual learning of the unexplored space. For instance, WPATH (d555), proposed by H. Wiener, captured graph topology, e.g., branching and size, and was introduced to the model in the earliest iteration.^39^ It is hypothesized that the number helped depict the general patterns of the introduced compounds, e.g., it is known that larger molecules tend to have higher log *IE* values.^40^ Nevertheless, this becomes less informative as the model needs more detailed descriptions of the dense chemical space.

On the other hand, the descriptor BCUTw.1l (d25), initially included in the model, became progressively less important. Its diminished informativeness could be attributed to the similar distribution between the explored and the unexplored space, especially when there was an increasing number of descriptors with potentially more considerable differences introduced to the model in the AL workflow.

AATS6s (d1115) was previously reported as one of the most significant descriptors in a random forest model for log *IE* prediction in positive mode LC/ESI/HRMS.^11,31^ Similarly, for the negative mode AATS5m (d1021), belonging to the same descriptor category, emerged as the most important descriptor in the current study (Figure S28). Different descriptors yielding the highest variable importance could result from the diverse mechanism of ionization as well as from the difference in chemical space coverage. Here, the distribution of AATS6s values for explored and unexplored chemical space largely overlapped, suggesting its low informativeness in chemical space exploration (Figure S43). Additionally, the relatively high correlation between AATS6s and AATS5m (d1021) also implies their similarity (Figure S44).

### Chemical Space Exploration to Improve the Performance of the IE Prediction Model

The observed difference in the predicted log *IE* values and quantification accuracy emphasized the importance of chemical space exploration before applying ML models to new chemical spaces. The concentration predictions were up to 2.2× different, which could affect the following conclusions, e.g., the selection of source materials for natural product extraction or risk assessment in environmental monitoring.

## Conclusions

In the current study, five different AL approaches and five different *n*_sample_ were compared for their ability to sample new chemical space that could improve log *IE* prediction accuracy using xgBoost ML models. It was discovered that higher diversity in the early stages of AL facilitated the efficiency of exploration, whereas considering informativeness, which represents the uncertainty of predictions, became more important in the later stages when the majority of the chemical space had been explored. The evaluation of the exploration was limited by a relatively small chemical space, where differences between larger *n*_sample_, such as 15 and 20, were not apparent due to the prompt increase in the proportion of the selected chemicals, which quickly covered the unexplored space. The lower diversity of the chemicals sampled from the unexplored space constrained the uncertainty-based AL approach, while the random sampling was able to efficiently explore the chemical space. As a result, operating a combined AL approach with sophisticated algorithms on a diverse unexplored space or conducting alternative AL strategies, such as cost-optimized strategies, could furnish a more comprehensive understanding of the AL process and the ML prediction-making procedure. Overall, independent of the sampling approaches, the prediction error dropped significantly after the first iteration. This advance highlights the influence of the chemical space coverage of the training set in ML training. To finalize, the findings indicated the potential of ML tools in assisting quantification in HRMS without analytical standards.

## Supplementary Material





## Notes

Methods of experimental data acquisition and supporting figures (PDF)
Table for PaDEL descriptors encoding (XLSX) (ZIP)
