# Supplementary material for: Active Learning Improves Ionization Efficiency Predictions and Quantification in Nontargeted LC/HRMS
Source: Anal Chem. 2025 Jun 13;97(25):13131–9. doi: 10.1021/acs.analchem.5c00816 (PMC12224155; doi:10.1021/acs.analchem.5c00816)
Supplement: Supplementary file 1 [file ac5c00816_si_001.pdf]

Active learning improves ionization efficiency predictions and quantification in non-targeted LC/HRMS

Wei-Chieh Wang,[a] Nahid Amini,[b] Carolin Huber,[c] Meelis Kull,[d] Anneli Kruve*,[a,e]

[a]Department of Materials and Environmental Chemistry, Stockholm University, Svante Arrhenius väg 16, 114 18 Stockholm, Sweden

[b]Oriflame Cosmetics, Scientific Research & Technology, Fleminggatan 14, 112 26 Stockholm, Sweden

[c] Department of Exposure Science, Helmholtz Centre for Environmental Research – UFZ, Permoserstr. 15, 04318 Leipzig, Germany

[d]Institute of Computer Science, University of Tartu, Narva mnt 18, 51009, Tartu, Estonia

[e]Department of Environmental Science, Stockholm University, Svante Arrhenius väg 8, 114 18 Stockholm, Sweden

Table of content

Method 5

Measurement of a reference standard mixture containing chemicals of environmental interest 5

Performance evaluation 6

Table 7

Table S1. Gradient profile during the measurement time used for data acquisition of the mixture of environmental contaminants. 7

Table S2. Ion source and parameters of the mass spectrometer 7

Table S3. MZmine parameters applied for data processing 8

Table S4. Information about the explored and the unexplored space in the space-separate cross-validation. 8

Table S5. PaDEL descriptors encoding 8

Table S6. Standard deviation (SD) of the $RMSE_{pooled}$ for different AL and nsample between 20 iterations and across 50 repetitions. 9

Figure 10

Figure S1. Correlation between the distance from the explored to the unexplored space and the prediction interval. 10

Figure S2. Clustering results from the *k*-means algorithm with the classification from (A) the dataset resources when the *k* = 3 and distribution of the superclasses from ClassyFire of the chemicals when (B) *k* = 5, (C) *k* = 15, and (D) *k* = 20. 12

Figure S3. Mean distance distribution for each cluster in the (A) clustering-based sampling and (B) mix sampling 13

Figure S4. Space-separated cross-validation for anticlustering and uncertainty-based AL with n_{sample} = 20. 13

Figure S5. Uncertainty-based AL for different sets of explored and unexplored space with different n_{sample}. 14

Figure S6. Model evaluation from the first to the 6th iteration with selected algorithms and n_{sample} = 5. 15

Figure S7. Model evaluation from the first to the 6th iteration with selected algorithms and n_{sample} = 5. 16

Figure S8. Model evaluation from the first to the 6th iteration with selected algorithms and n_{sample} = 10 17

Figure S9. Model evaluation from the first to the 6th iteration with selected algorithms and n_{sample} = 15 18

Figure S10. Model evaluation from the first to the 6th iteration with selected algorithms and n_{sample} = 20 19

Figure S11. Chemical space exploration in the early stages of AL (the 1st to the 7th iteration) when n_{sample} = 5. 20

Figure S12. Chemical space exploration in the early stages of AL (the 1st to the 7th iteration) when n_{sample} = 10. 21

Figure S13. Chemical space exploration in the early stages of AL (the 1st to the 7th iteration) when n_{sample} = 15. 22
Figure S14. Chemical space exploration in the early stages of AL (the 1st to the 7th iteration) when n_{sample} = 20. 23
Figure S15. Changes of variable importance in AL iteration for random sampling with n_{sample} = 1. 24
Figure S16. Changes of variable importance in AL iteration for random sampling with n_{sample} = 5. 25
Figure S17. Changes of variable importance in AL iteration for random sampling with n_{sample} = 10. 26
Figure S18. Changes of variable importance in AL iteration for random sampling with n_{sample} = 15. 27
Figure S19. Changes of variable importance in AL iteration for random sampling with n_{sample} = 20. 28
Figure S20. Changes of variable importance in AL iteration for uncertainty-based sampling with n_{sample} = 1. 29
Figure S21. Changes of variable importance in AL iteration for uncertainty-based sampling with n_{sample} = 5. 30
Figure S22. Changes of variable importance in AL iteration for uncertainty-based sampling with n_{sample} = 10. 31
Figure S23. Changes of variable importance in AL iteration for uncertainty-based sampling with n_{sample} = 15. 32
Figure S24. Changes of variable importance in AL iteration for uncertainty-based sampling with n_{sample} = 20. 33
Figure S25. Changes of variable importance in AL iteration for clustering-based sampling with n_{sample} = 1. 34
Figure S26. Changes of variable importance in AL iteration for clustering-based sampling with n_{sample} = 5. 35
Figure S27. Changes of variable importance in AL iteration for clustering-based sampling with n_{sample} = 10. 36
Figure S28. Changes of variable importance in AL iteration for clustering-based sampling with n_{sample} = 15. 37
Figure S29. Changes of variable importance in AL iteration for clustering-based sampling with n_{sample} = 20. 38
Figure S30. Changes of variable importance in AL iteration for anti-clustering-based sampling with n_{sample} = 5. 39
Figure S31. Changes of variable importance in AL iteration for anti-clustering-based sampling with n_{sample} = 10. 40
Figure S32. Changes of variable importance in AL iteration for anti-clustering-based sampling with n_{sample} = 15. 41

Figure S33. Changes of variable importance in AL iteration for anti-clustering-based sampling with n_{sample} = 20. 42

Figure S34. Changes of variable importance in AL iteration for mix sampling with n_{sample} = 5. .. 43

Figure S35. Changes of variable importance in AL iteration for mix sampling with n_{sample} = 10. 44

Figure S36. Changes of variable importance in AL iteration for mix sampling with n_{sample} = 15. 45

Figure S37. Changes of variable importance in AL iteration for mix sampling with n_{sample} = 20. 46

Figure S38. The number of descriptors in each iteration for various algorithms 47

Figure S39. Proportions of the descriptor types for PaDEL descriptors[2] 47

Figure S40. Demonstration cases of suggestions from random approach with insufficient diversity. (A) n_{sample} = 10, the 14th iteration, and the 1st repetition. (B) n_{sample} = 5, the 13th iteration, and the 2nd repetition. 48

Figure S41. AL performance evaluation with selected algorithms compared to cost-based sampling 48

Figure S42. Performance of the ML log*IE* prediction models on 20% of the explored space with random sampling for 15 repetitions. The x-axis was the proportion of the labeled compounds over the compounds in the whole unexplored space with each iteration step. Each point in the plots represented the average result from all repetitions, and the color ranges were inferred from the SEM. 49

Figure S43. Distribution comparison between the explored space and unexplored space for descriptors in interest 50

Figure S44. Correlation between the explored space and unexplored space for descriptors AATS5m (d1021) and AATS6s (d1115) 50

Reference 51

Method

Measurement of a reference standard mixture containing chemicals of environmental interest

Environmental contaminants

The LC system consisted of an UltiMate LPG-3000 dual pump, a WPS-3000 autosampler, and a TCC-3000 SD column oven (Thermo Scientific). A Kinetex 2.6 µm EVO C18 (50 × 2.1 mm) column with a pre-column (C18 EVO 5 × 2.1 mm) and an inline filter was used at 40°C to perform chromatographic separation at a flow rate of 300 µL/min. A gradient of water, methanol (both containing 0.1% formic acid), and a mixture of isopropanol and acetone (50:50) for the final rinsing step without injection to the MS were used. The entire gradient profile during measurement is summarized in **Table S1**. Measurement was performed on a QExactive Plus (Thermo Scientific) with a HESI ion source in negative ionization. Full scan experiment (100-1500 m/z) at a nominal resolving power of 70,000 (referenced to m/z 200) and data-independent MS/MS experiments at a nominal resolving power of 35,000 with 12 different isolation windows were applied for identification. Further ion source and acquisition parameters can be found in the Supporting information, **Table S2**. The injection volume was set to 100 µL. A reference mixture containing the reference mixture in the concentration levels 1, 2, 5, 10, 20, 50, 100, 200, 500, 1000, 2000, and 5000 ng/L, was prepared in a water/ methanol solution (70:30 v/v) and run in increasing order. A full list of all chemicals in the mixture can be found on Zenodo (https://zenodo.org/records/3365550).

Raw data processing was performed using Mzmine version 2.38[1], including peak picking, alignment, and gap filling. All parameters applied are summarized in **Table S3**.

Natural products

Natural compounds from the Enzo SCREEN-WELL® Natural Product library were supplied at an initial concentration of 2 mg/mL in dimethyl sulfoxide and were serially diluted using a 50% ethanol solution to final concentrations of 100.00, 50.00, 10.00, 5.00, 2.50, 1.00, 0.50, 0.10, 0.05, and 0.01 µg/mL. Calibration curves were generated for five compounds from the Natural Product Library: epicatechin, galangin, luteolin-3,7-diglucoside, pinocembrin, and catechin. Liquiritigenin (Sigma-Aldrich) was used as an internal standard at a final concentration of 10 µg/mL in all samples.

All the samples were analyzed using an ACQUITY UPLC I-Class PLUS coupled with an ACQUITY RDa Detector (Waters™). Chromatographic separation was achieved on an ACQUITY Premier BEH C18 column (1.7 µm, 2.1 × 100 mm) maintained at 50°C. The mobile phase consisted of water with 0.1% formic acid (phase A) and acetonitrile with 1% formic acid (phase B). The flow rate was set at 0.4 mL/min, with a gradient starting at 0% B, increasing to 60% B at 30 minutes, and reaching 100% B at 45 minutes. Each analysis utilized an injection volume of 3 µL.

Ionization was performed using negative ESI. The RDa detector operated in full-scan mode (50 – 2000 m/z) at a scan rate of 5 Hz, with and without fragmentation. For fragmentation, the cone voltage was set between 100 – 190 V, while non-fragmented analysis was performed at a cone voltage of 40 V.

An extract of Alpinia officinarum rhizome was prepared by weighing 1 g of plant material and extracting it with 20 mL of water. The mixture was subjected to sonication at room temperature for 2 hours, followed by centrifugation at 2000g for 5 minutes. The supernatant was filtered through a Büchner funnel using Whatman filters of grades 1 and 4 and subsequently freeze-dried for approximately 4 days.

A solution of Alpinia officinarum extract at a concentration of 8.68 µg/mL, containing the internal standard liquiritigenin (10 µg/mL), was prepared for the quantification of the identified compounds.

Performance evaluation

Space-separated cross-validation was conducted to validate the performance of different AL strategies. To this end, different explored and unexplored space pairs were set, and AL strategies, anticlustering, and uncertainty-based sampling were used. For anticlustering AL, nsample was set as 20, while nsample equal to 1, 5, 10, 15, and 20 were implemented. The detailed information about the space pairs is summarized in **Table S4**. The training set was acquired by randomly taking 80% of the unique compounds in the whole unexplored space. Only one instance would be randomly selected if more than one measurement was executed for the same compound while making the training and test sets. The whole validation included 20 AL iterations and 50 iterations. SEM was computed to determine the width of the colored ribbon for each strategy.

Table

Table S1. Gradient profile during the measurement time used for data acquisition of the mixture of environmental contaminants.

Gradient time (min)	Mobile phase B (MeOH) (%)
0	5
1	5
13	100
24	100

Table S2. Ion source and parameters of the mass spectrometer

Parameters	Values
Sheath gas flow rate	45 a.u.
Aux gas flow rate	3 a.u.
Sweep gas flow rate	3.5 KV
Capillary	300°C
Aux gas heater	300°C
Resolution MS1	70,000
Mass range	100 - 1500
Ion accumulation per scan event	120 ms

Table S3. MZmine[1] parameters applied for data processing

Calculation step	Parameters	Values
Mass detection	Noise level	5000
ADAP Chromatogram builder	Min consec. scans	8
	Min intensity	10000
	Min. abs. height	5000
	m/z tolerance	0.001
Smoothing	Filter width	7
Alignment	method	Join Aligner
	m/z tolerance	0.001
	Weight for m/z	70
	Weight for RT	30
	Isotope m/z tolerance	0.001 (5 ppm)
	Min absolute score intensity	50000
	Min score	0.7
Custom DB Search	m/z tolerance	0.001 (7 ppm)
	RT tolerance	0.6
Gapfilling	Intensity tolerance	0.3
	m/z tolerance	0.001 (7 ppm)
	RT tolerance	0.15

Table S4. Information about the explored and the unexplored space in the space-separate cross-validation.

Explored space	Unique chemicals	Unexplored space	Unique chemicals	Total number of instances
Non-PFAS	81	NP + EC + PCB + PFAS	369	369
Natural products (NP)	217	Non-PFAS + EC + PCB + PFAS	233	1161
Environmental contaminants (EC)	102	Non-PFAS + NP + PCB + PFAS	348	1276
PCB + PFAS	50	Non-PFAS + NP + EC	400	1328

Table S5. PaDEL descriptors[2] encoding

Supplementary file: df_PaDEL_ID.csv

Table S6. Standard deviation (SD) of the $RMSE_{pooled}$ for different AL and nsample between 20 iterations and across 50 repetitions.

Algorithm	n_{sample}	SD_{RMSE}	Algorithm	n_{sample}	SD_{RMSE}	Algorithm	n_{sample}	SD_{RMSE}
Random	1	[0.016-0.030]	Uncertainty-based	1	[0.018-0.024]	Clustering-based	1	[0.020-0.047]
	5	[0.018-0.028]		5	[0.017-0.025]		5	[0.018-0.030]
	10	[0.017-0.024]		10	[0.016-0.024]		10	[0.017-0.028]
	15	[0.017-0.024]		15	[0.017-0.025]		15	[0.017-0.027]
	20	[0.014-0.025]		20	[0.017-0.023]		20	[0.017-0.025]
Anticlustering	5	[0.018-0.028]	Mix	5	[0.017-0.030]			
	10	[0.017-0.024]		10	[0.017-0.025]			
	15	[0.017-0.024]		15	[0.018-0.027]			
	20	[0.016-0.023]		20	[0.017-0.024]			

Figure

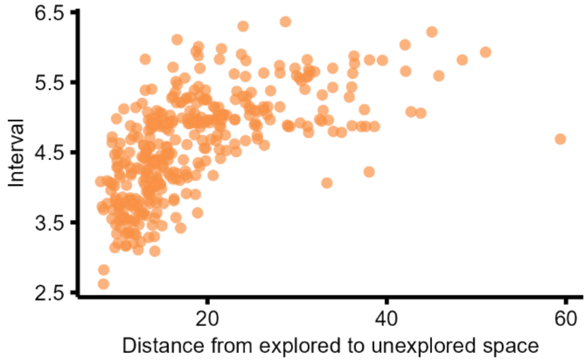

Figure S1. Correlation between the distance from the explored to the unexplored space and the prediction interval.

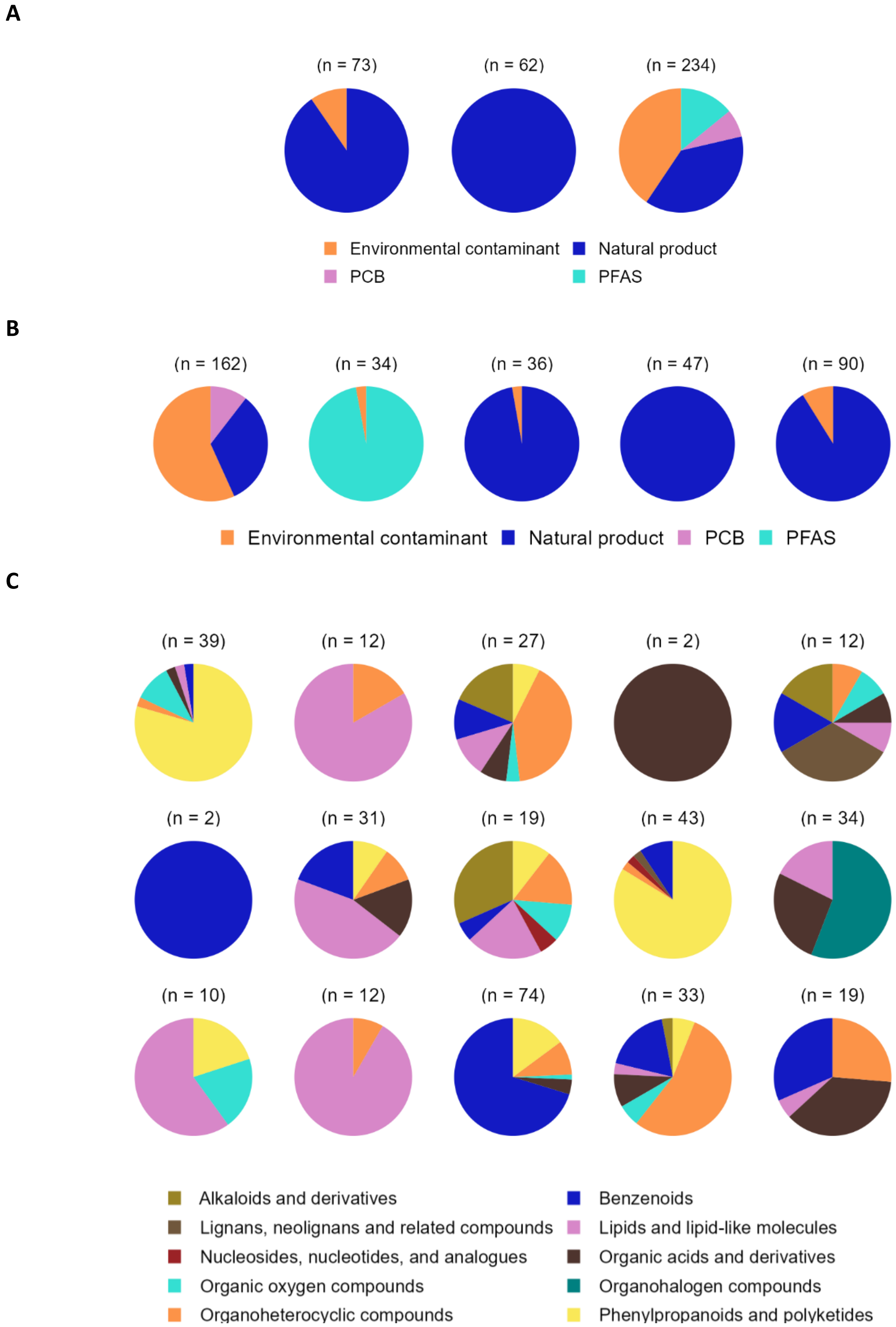
A
(n = 73)
(n = 62)
(n = 234)
Environmental contaminant
Natural product
PCB
PFAS
B
(n = 162)
(n = 34)
(n = 36)
(n = 47)
(n = 90)
Environmental contaminant
Natural product
PCB
PFAS
C
(n = 39)
(n = 12)
(n = 27)
(n = 2)
(n = 12)
(n = 2)
(n = 31)
(n = 19)
(n = 43)
(n = 34)
(n = 10)
(n = 12)
(n = 74)
(n = 33)
(n = 19)
Alkaloids and derivatives
Benzenoids
Lignans, neolignans and related compounds
Lipids and lipid-like molecules
Nucleosides, nucleotides, and analogues
Organic acids and derivatives
Organic oxygen compounds
Organohalogen compounds
Organoheterocyclic compounds
Phenylpropanoids and polyketides

D

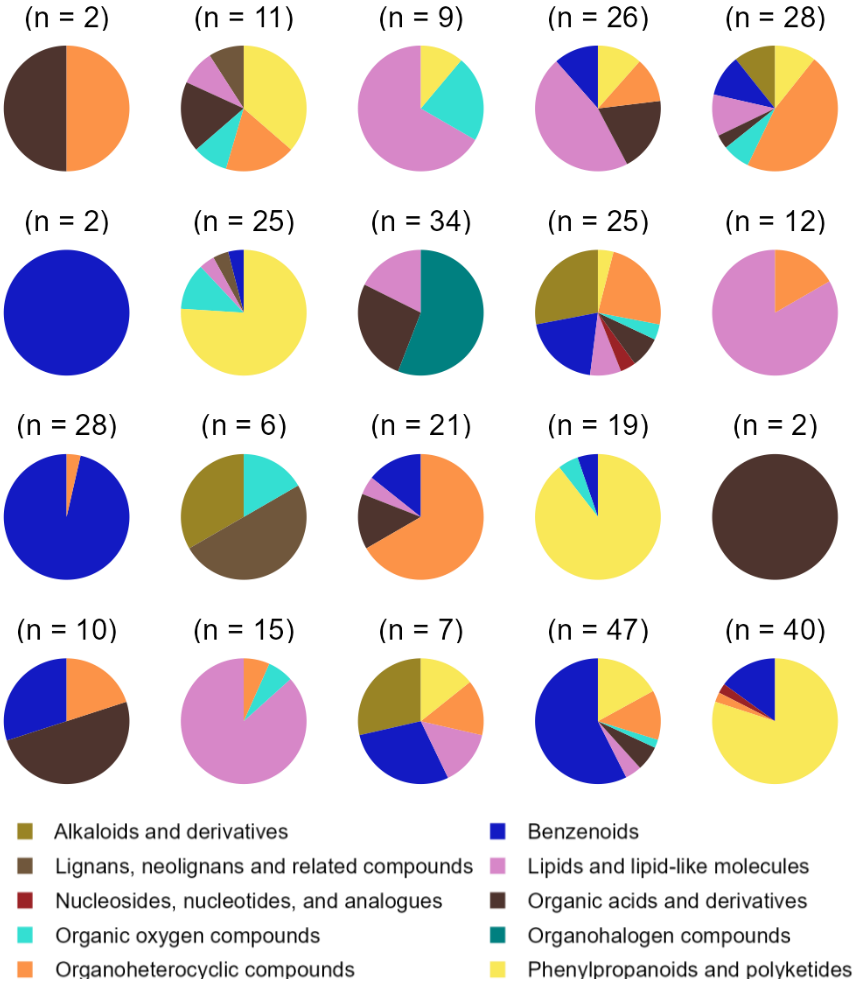

Figure S2. Clustering results from the *k*-means algorithm with the classification from (A) the dataset resources when the $k = 3$ and distribution of the superclasses from ClassyFire[3] of the chemicals when (B) $k = 5$, (C) $k = 15$, and (D) $k = 20$.

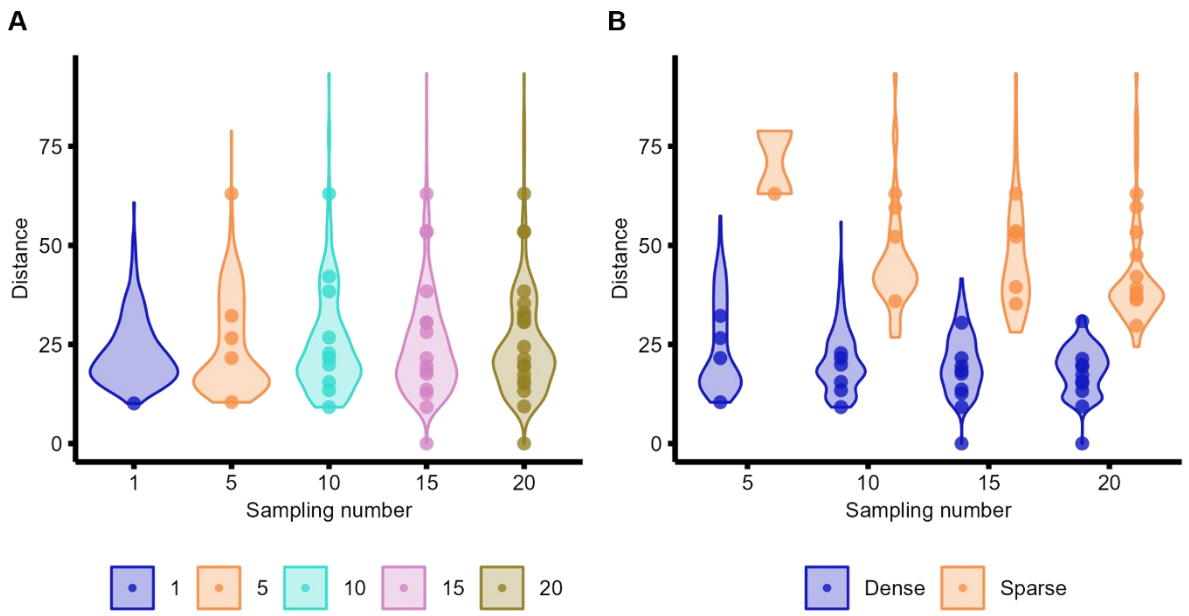

Figure S3. Mean distance distribution for each cluster in the (A) clustering-based sampling and (B) mix sampling.

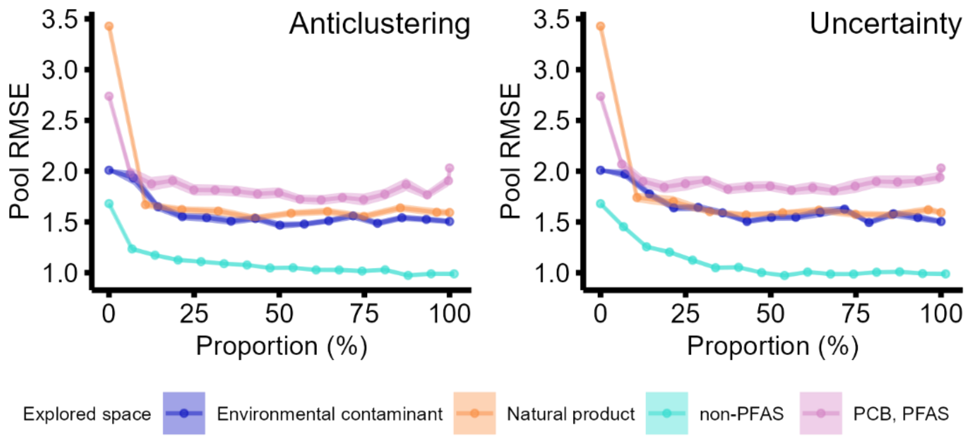

Figure S4. Space-separated cross-validation for anticlustering and uncertainty-based AL with n_{sample} = 20.

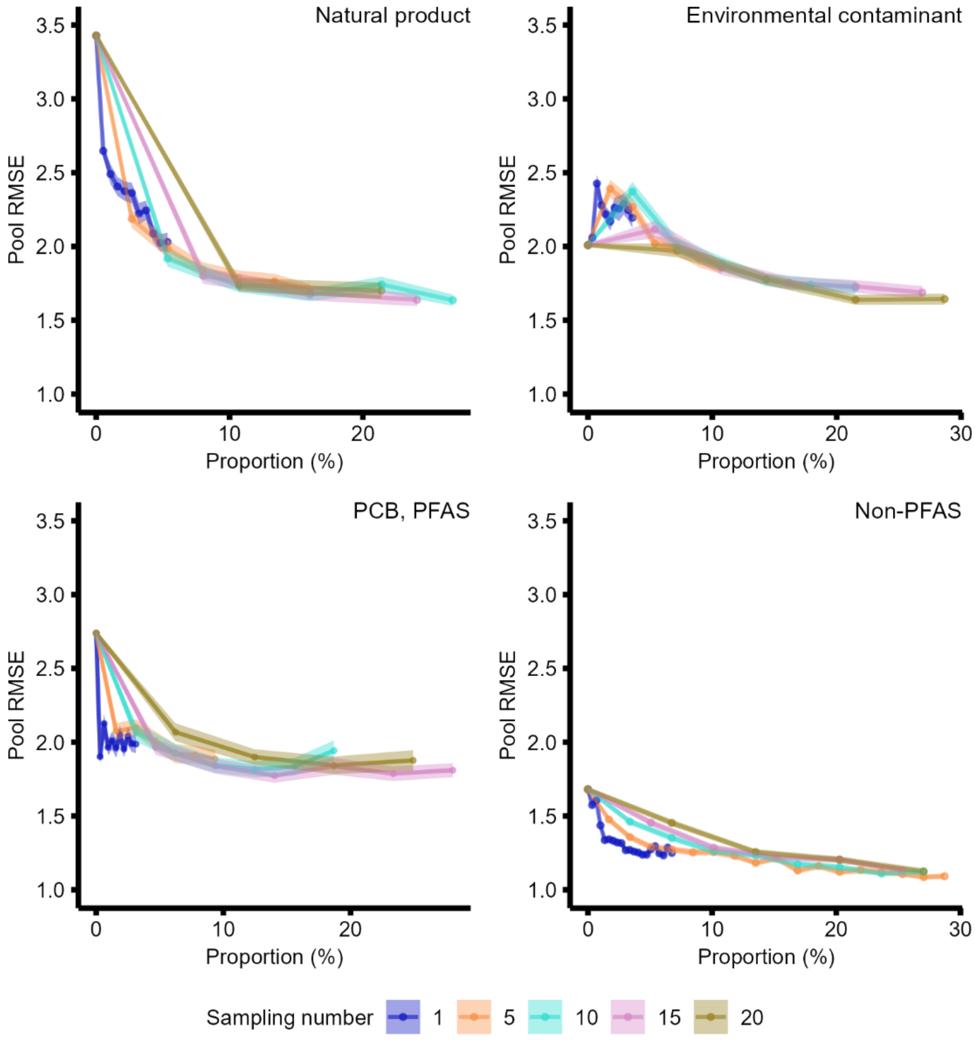

Figure S5. Uncertainty-based AL for different sets of explored and unexplored space with different n_{sample}.

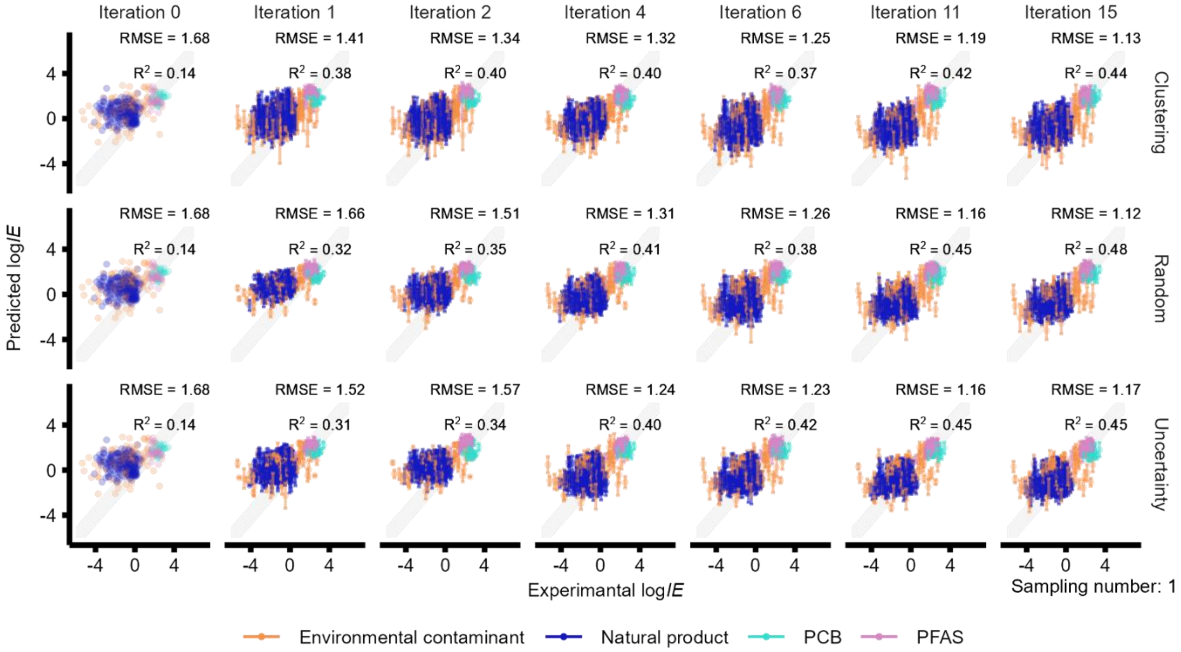

Figure S6. Model evaluation from the first to the 6th iteration with selected algorithms and n_{sample} = 5.

Each point in the plot represented the average of the test sets from the 50 repetitions. The grey region marked the ideal log*IE* predictions ±1 log*IE* unit.

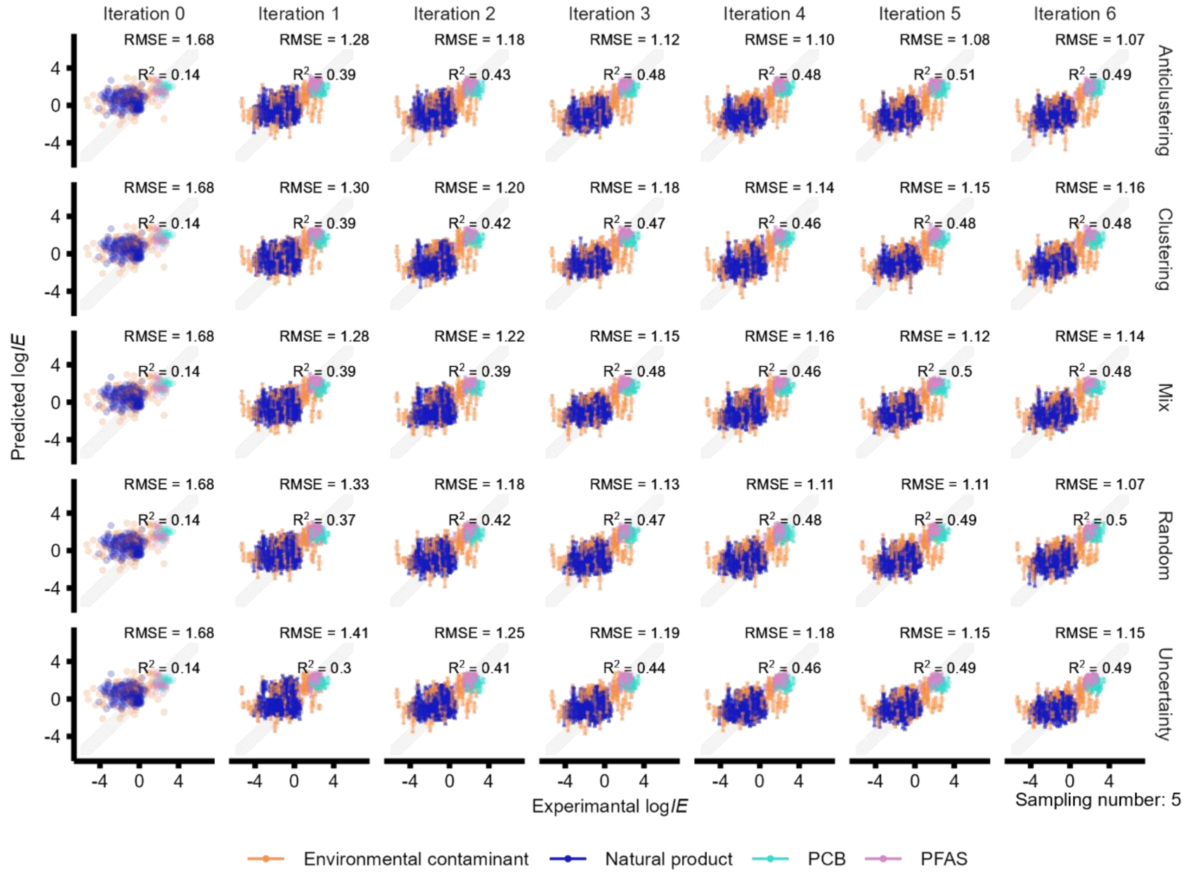

Figure S7. Model evaluation from the first to the 6th iteration with selected algorithms and n_{sample} = 5.

Each point in the plot represented the average of the test sets from the 50 repetitions. The grey region marked the ideal log*IE* predictions ±1 log*IE* unit.

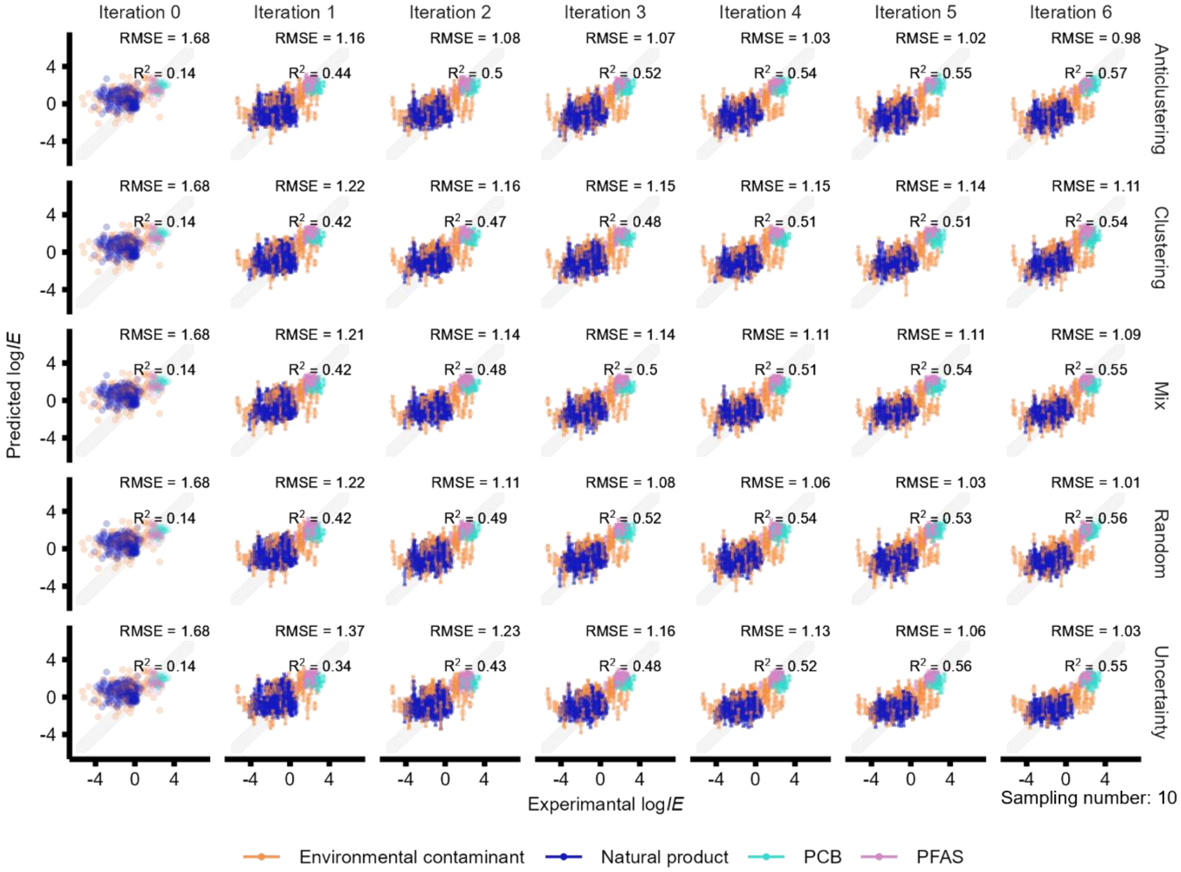

Figure S8. Model evaluation from the first to the 6th iteration with selected algorithms and n_{sample} = 10

Each point in the plot represented the average of the test sets from the 50 repetitions. The grey region marked the ideal logIE predictions ±1 logIE unit.

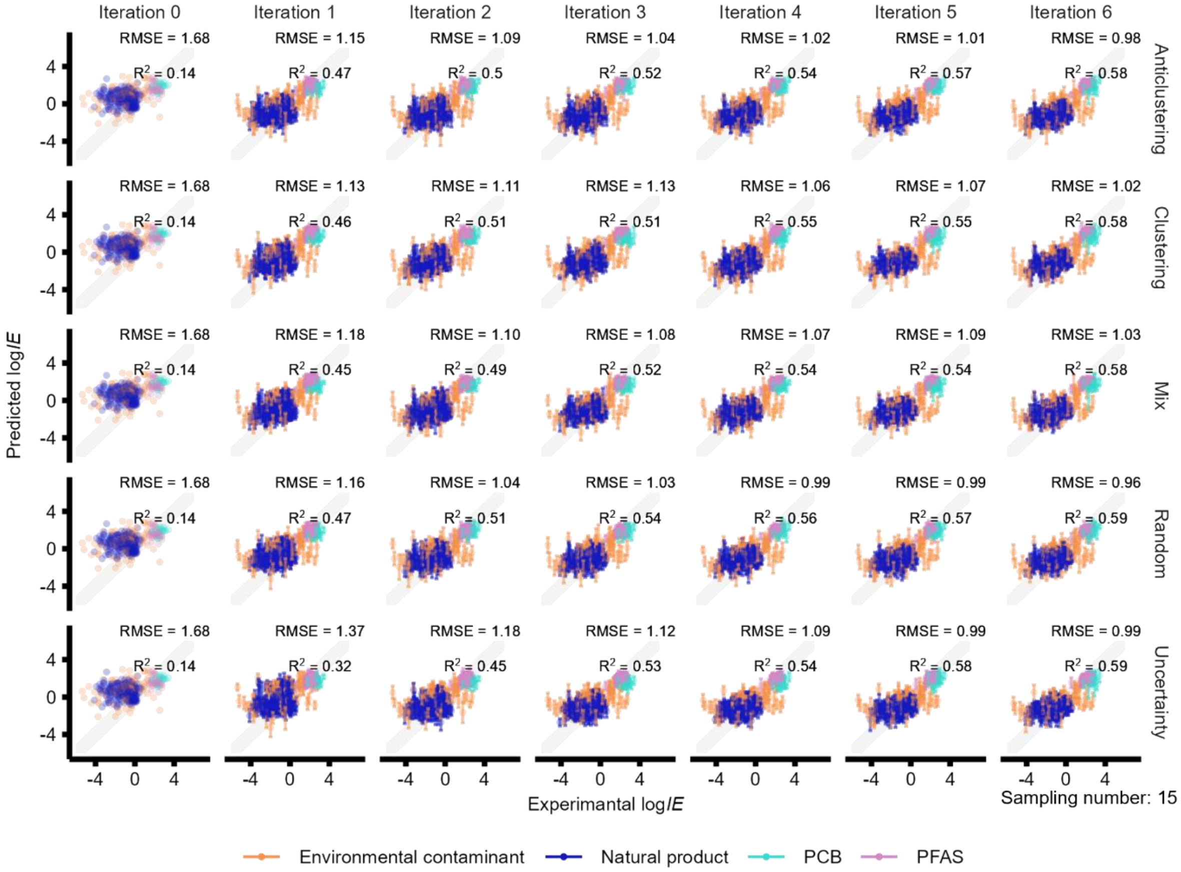

Figure S9. Model evaluation from the first to the 6th iteration with selected algorithms and n_{sample} = 15

Each point in the plot represented the average of the test sets from the 50 repetitions. The grey region marked the ideal logIE predictions ±1 logIE unit.

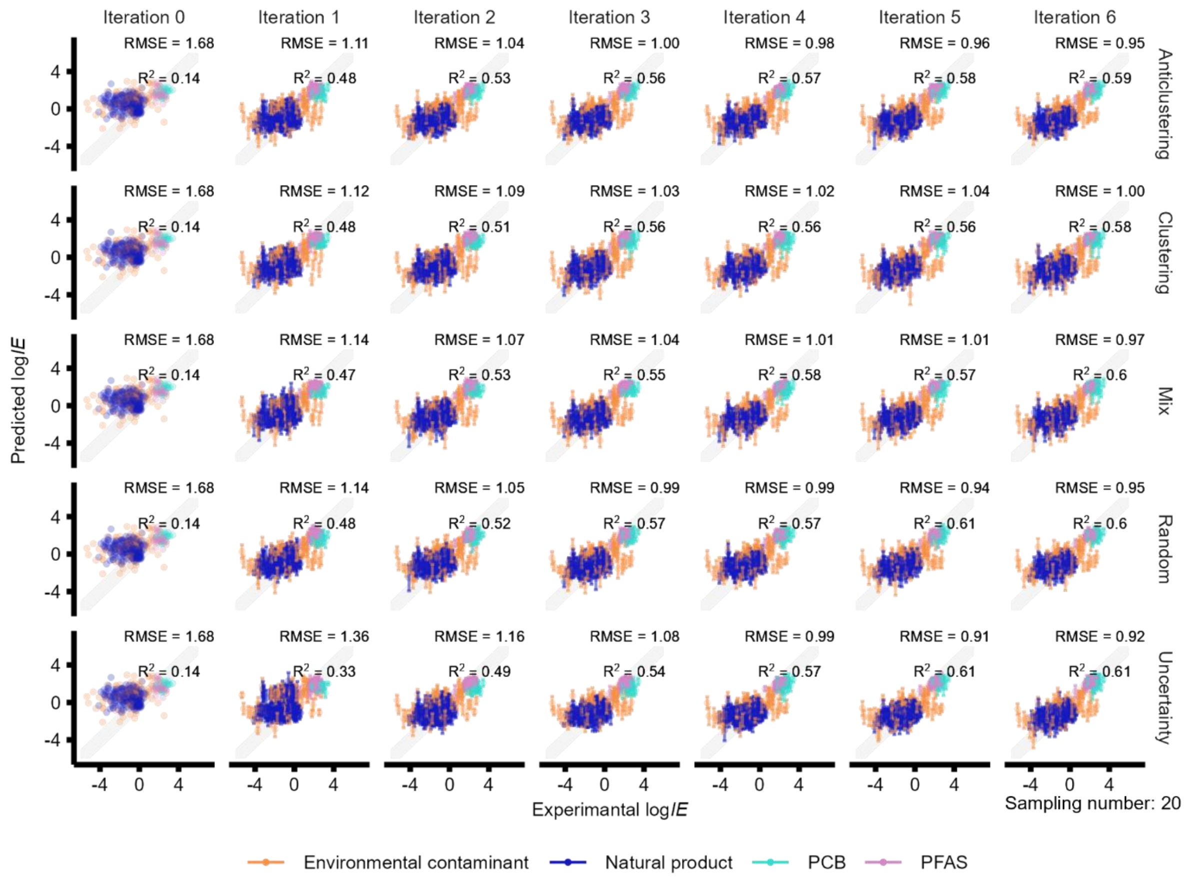

Figure S10. Model evaluation from the first to the 6th iteration with selected algorithms and n_{sample} = 20

Each point in the plot represented the average of the test sets from the 50 repetitions. The grey region marked the ideal logIE predictions ±1 logIE unit.

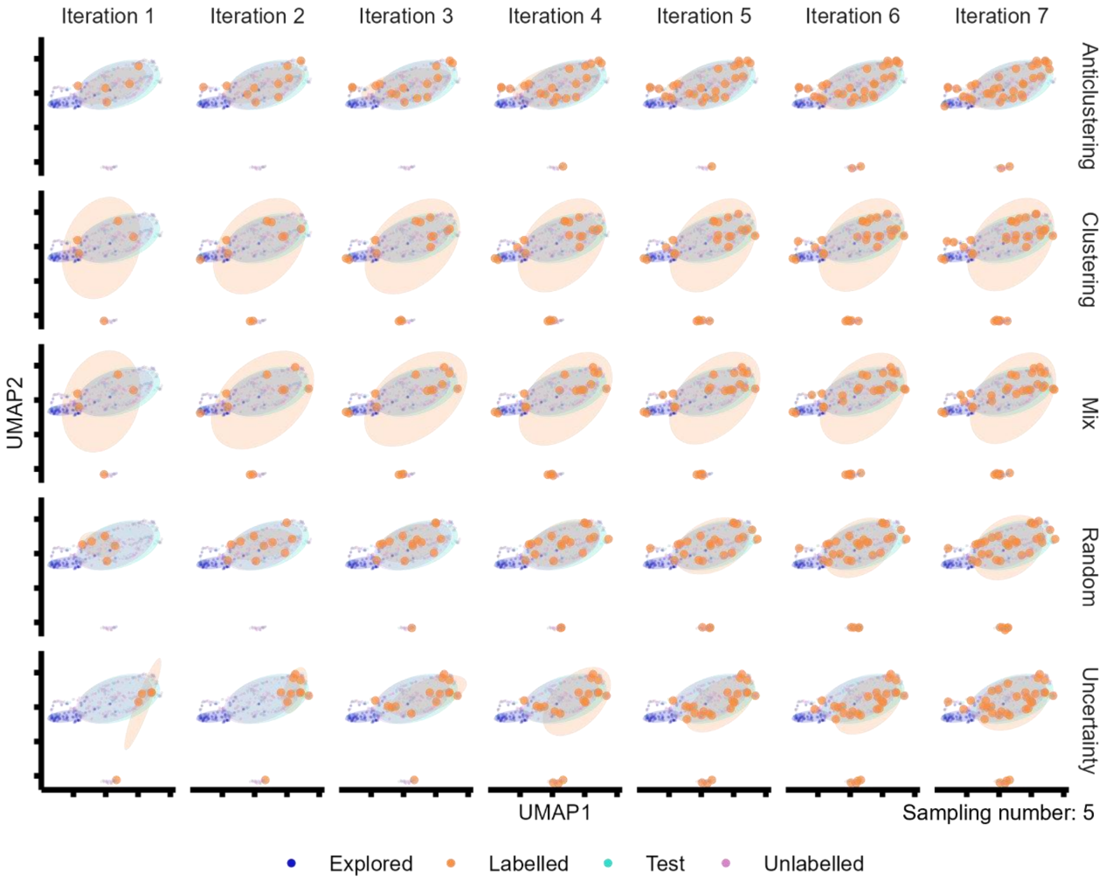

Figure S11. Chemical space exploration in the early stages of AL (the 1st to the 7th iteration) when n_{sample} = 5.

The first repetition was selected to be the representative for the evaluation. Parameters for ellipse computation: t-distribution and level equal 0.68. Highly overlapping areas between the testing points and the unlabelled points indicate a good representativeness of the target chemical space (test set).

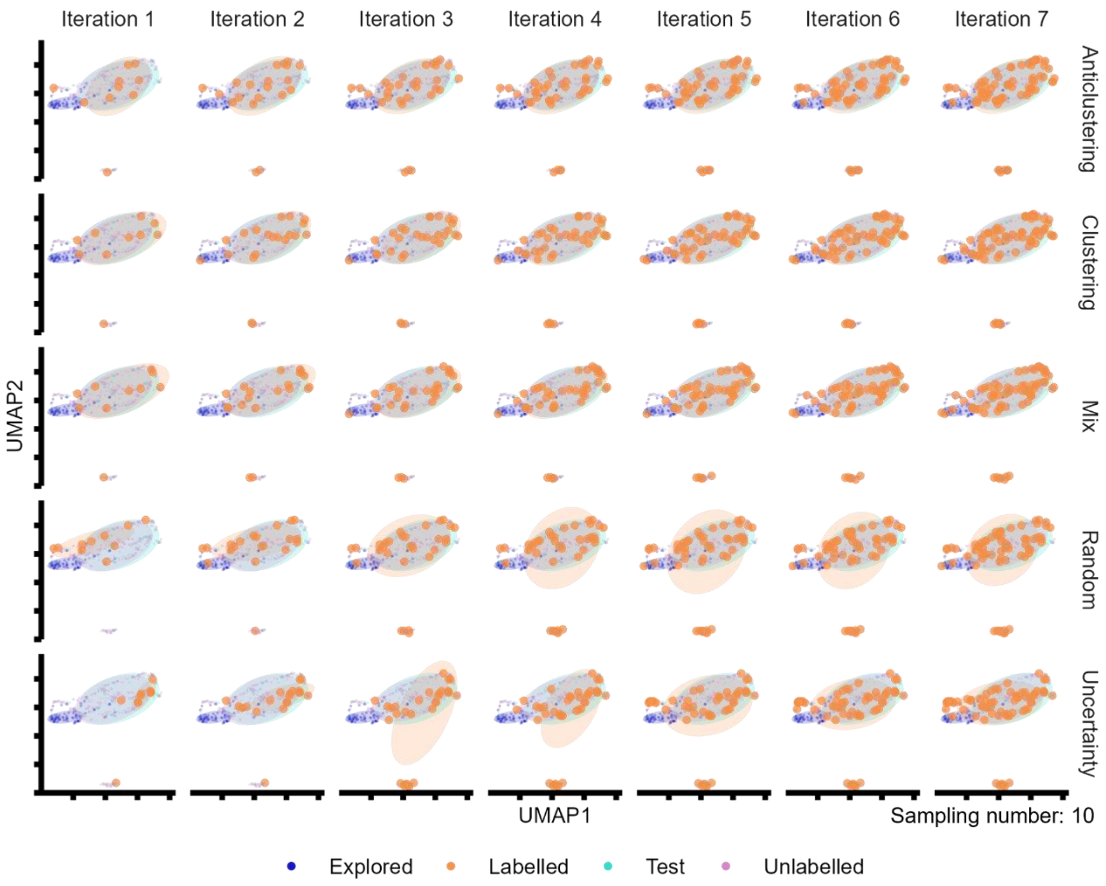

Figure S12. Chemical space exploration in the early stages of AL (the 1st to the 7th iteration) when n_{sample} = 10.

The first repetition was selected to be the representative for the evaluation. Parameters for ellipse computation: t-distribution and level equal 0.68. Highly overlapping areas between the testing points and the unlabelled points indicate a good representativeness of the target chemical space (test set).

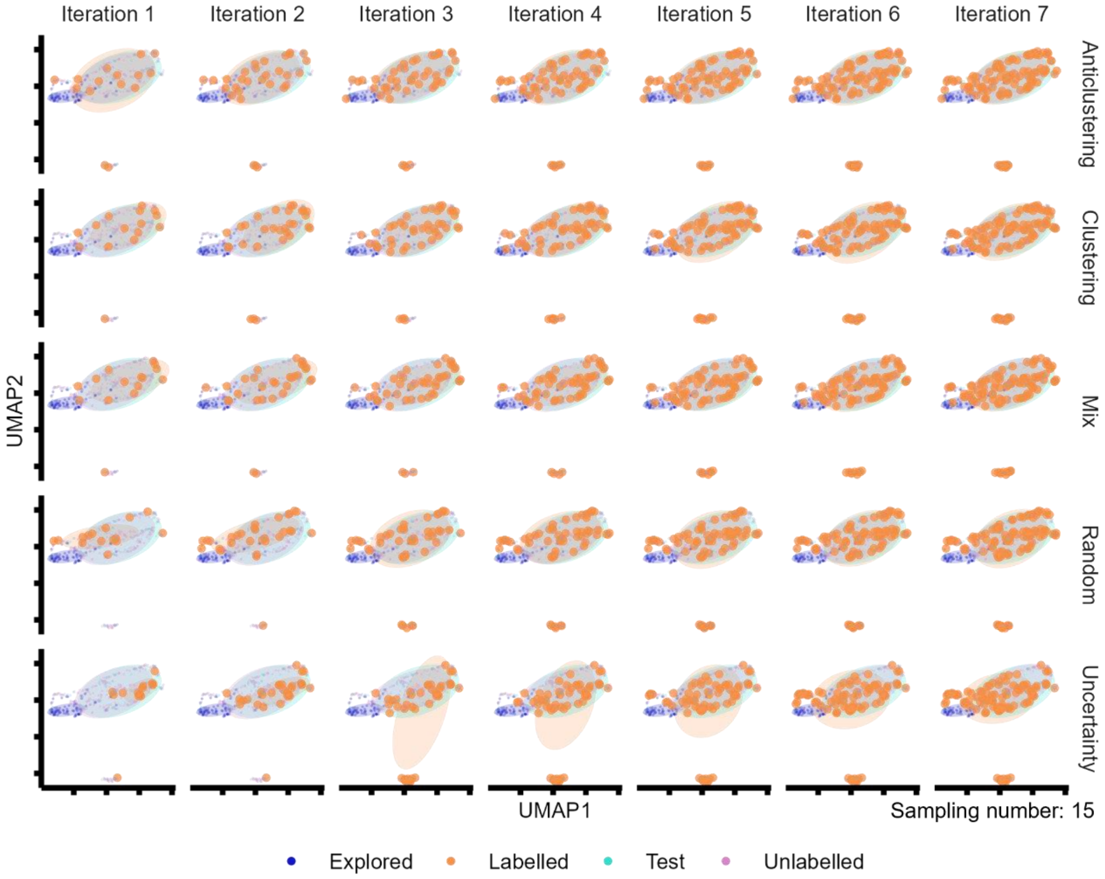

Figure S13. Chemical space exploration in the early stages of AL (the 1st to the 7th iteration) when n_{sample} = 15.

The first repetition was selected to be the representative for the evaluation. Parameters for ellipse computation: t-distribution and level equal 0.68. Highly overlapping areas between the testing points and the unlabelled points indicate a good representativeness of the target chemical space (test set).

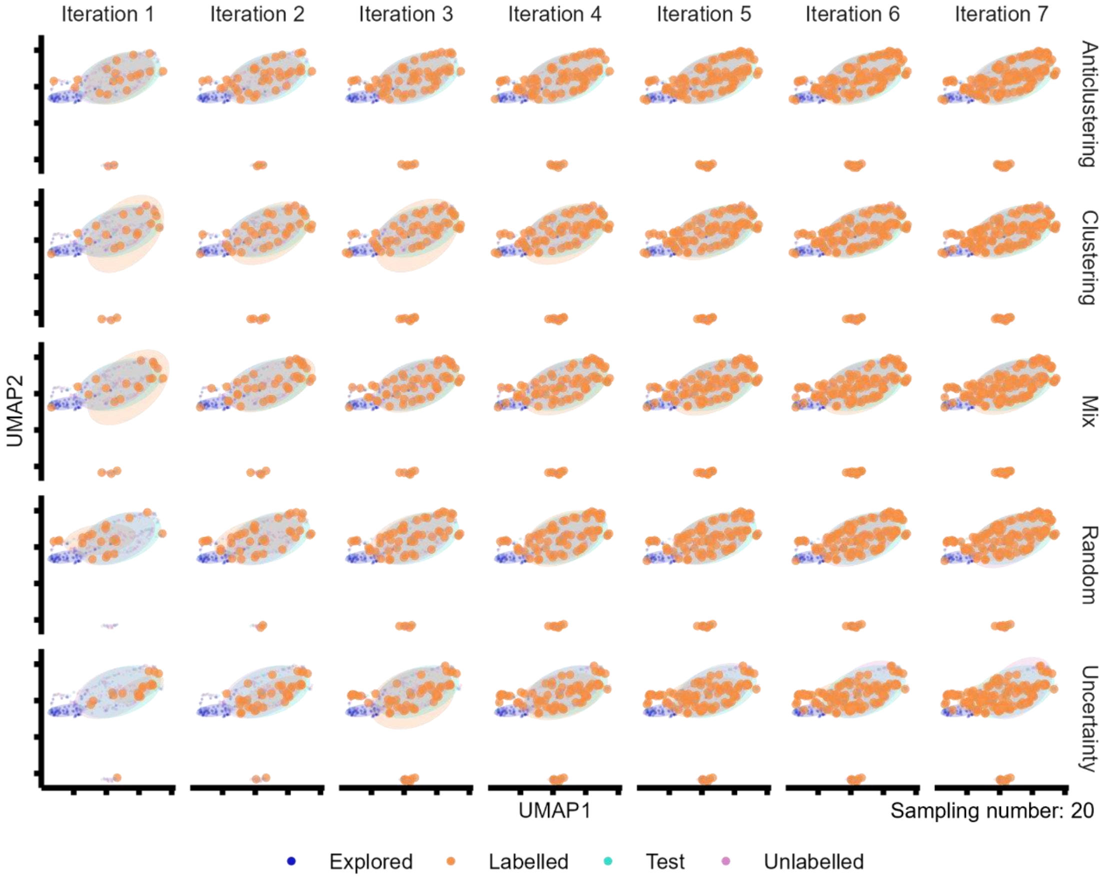

Figure S14. Chemical space exploration in the early stages of AL (the 1st to the 7th iteration) when n_{sample} = 20.

The first repetition was selected to be the representative for the evaluation. Parameters for ellipse computation: t-distribution and level equal 0.68. Highly overlapping areas between the testing points and the unlabelled points indicate a good representativeness of the target chemical space (test set).

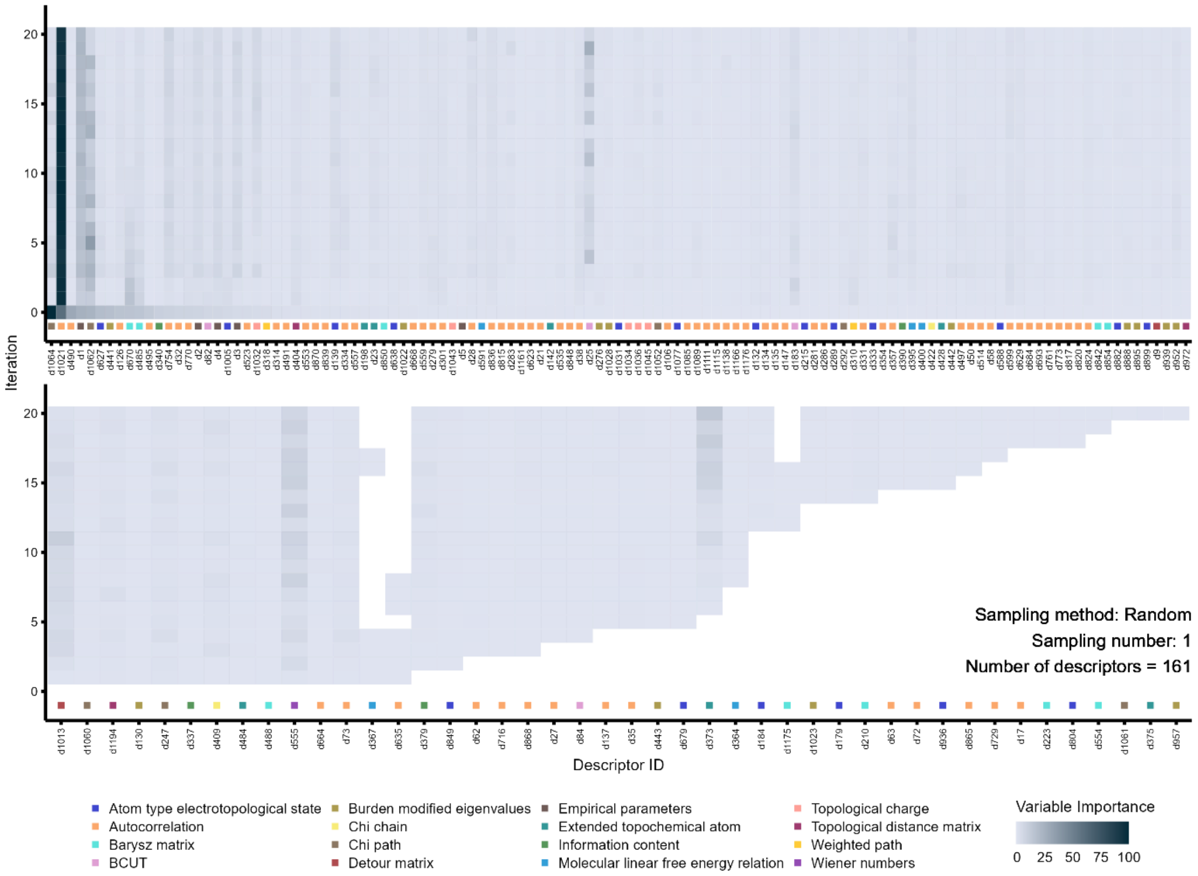

Figure S15. Changes of variable importance in AL iteration for random sampling with n_{sample} = 1.

All descriptors were encoded from d1 to d1225 to simplify the presentation (**Table S5**). The mean variable importance was evaluated overall by 50 repetitions for each iteration after replacing the NA values with zeros. Variances for the original model before AL were shown as the zeroth iteration.

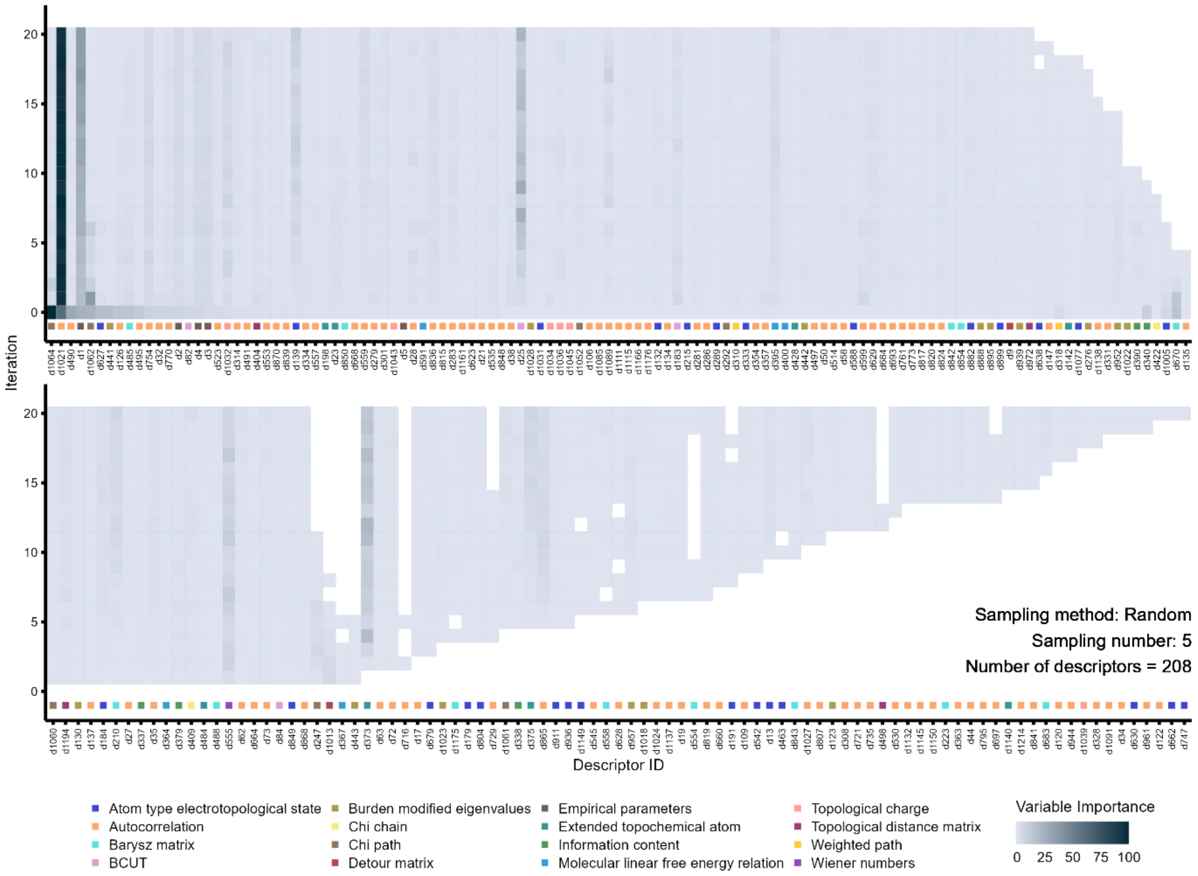

Figure S16. Changes of variable importance in AL iteration for random sampling with n_{sample} = 5.

All descriptors were encoded from d1 to d1225 to simplify the presentation (**Table S5**). The mean variable importance was evaluated overall by 50 repetitions for each iteration after replacing the NA values with zeros. Variances for the original model before AL were shown as the zeroth iteration.

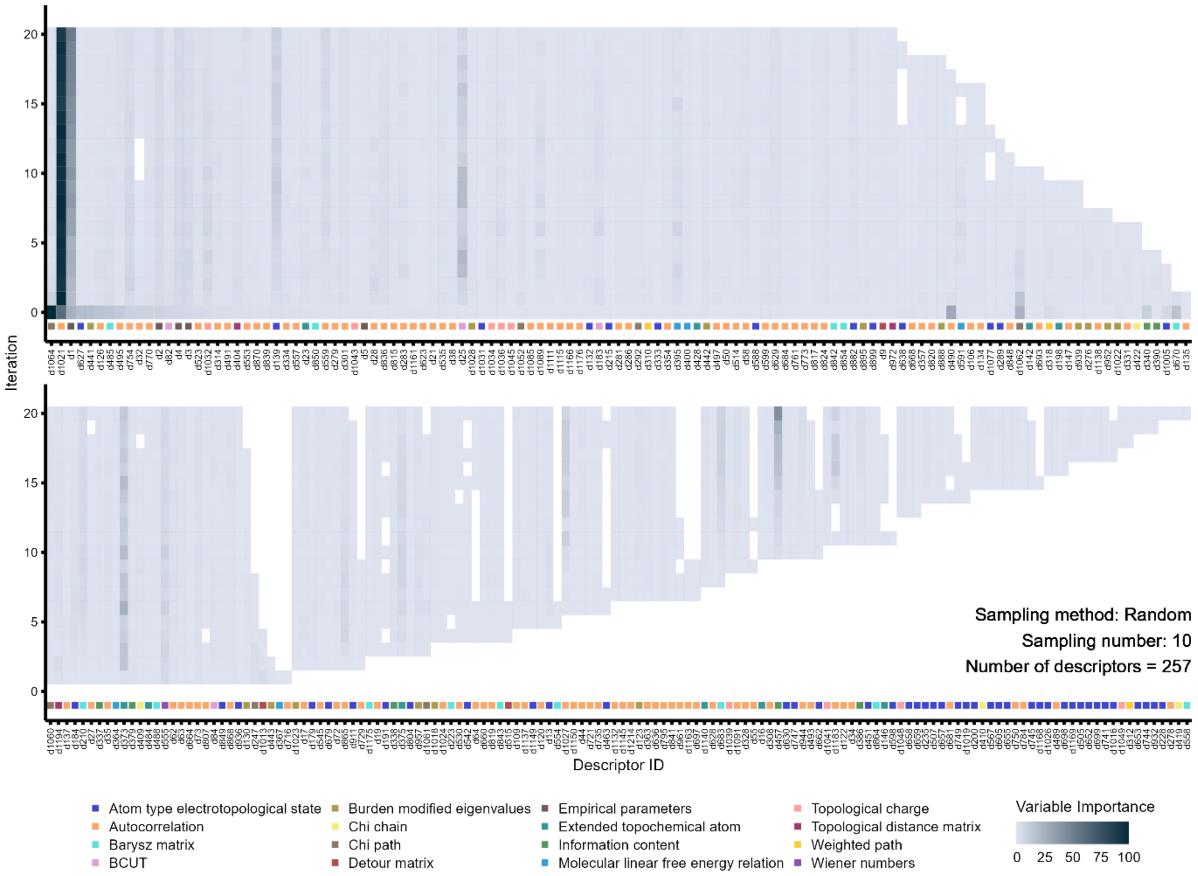

Figure S17. Changes of variable importance in AL iteration for random sampling with n_{sample} = 10.

All descriptors were encoded from d1 to d1225 to simplify the presentation (**Table S5**). The mean variable importance was evaluated overall by 50 repetitions for each iteration after replacing the NA values with zeros. Variances for the original model before AL were shown as the zeroth iteration.

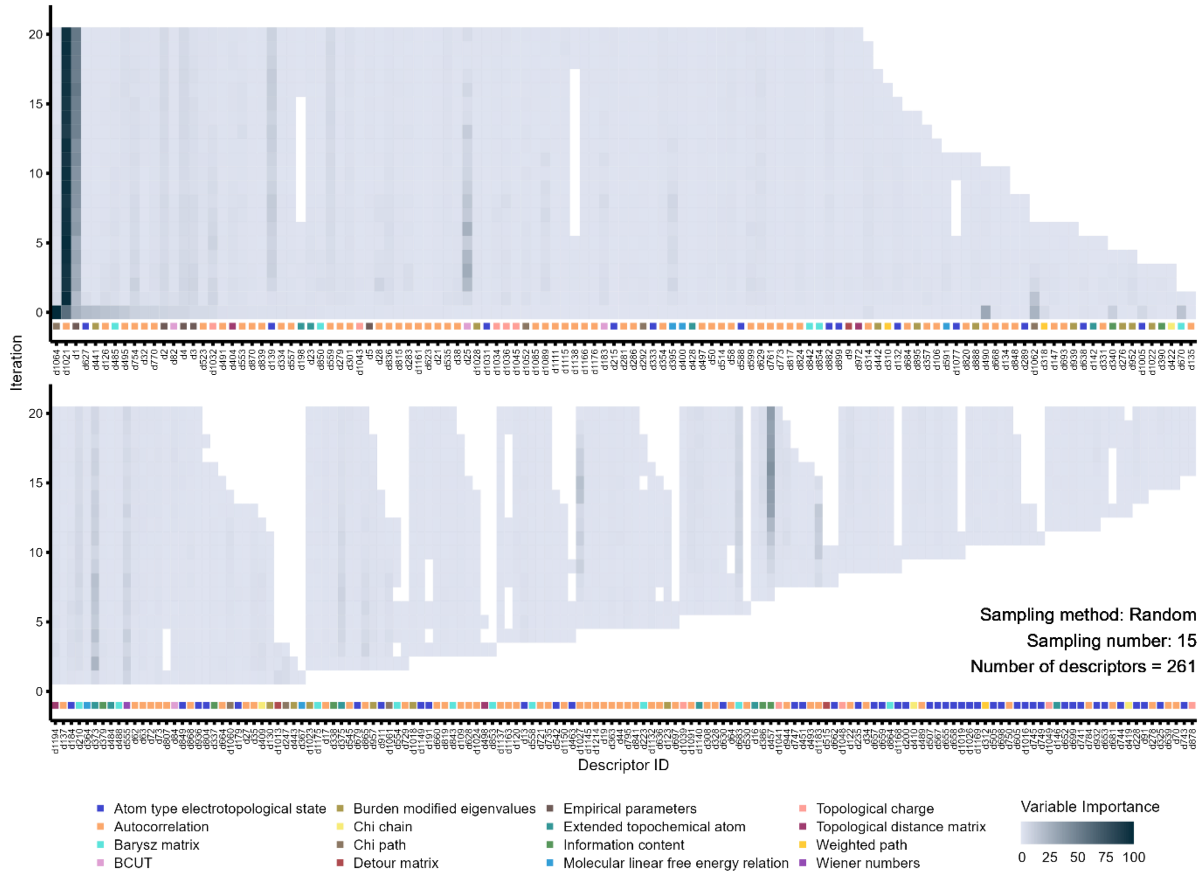

Figure S18. Changes of variable importance in AL iteration for random sampling with n_{sample} = 15.

All descriptors were encoded from d1 to d1225 to simplify the presentation (**Table S5**). The mean variable importance was evaluated overall by 50 repetitions for each iteration after replacing the NA values with zeros. Variances for the original model before AL were shown as the zeroth iteration.

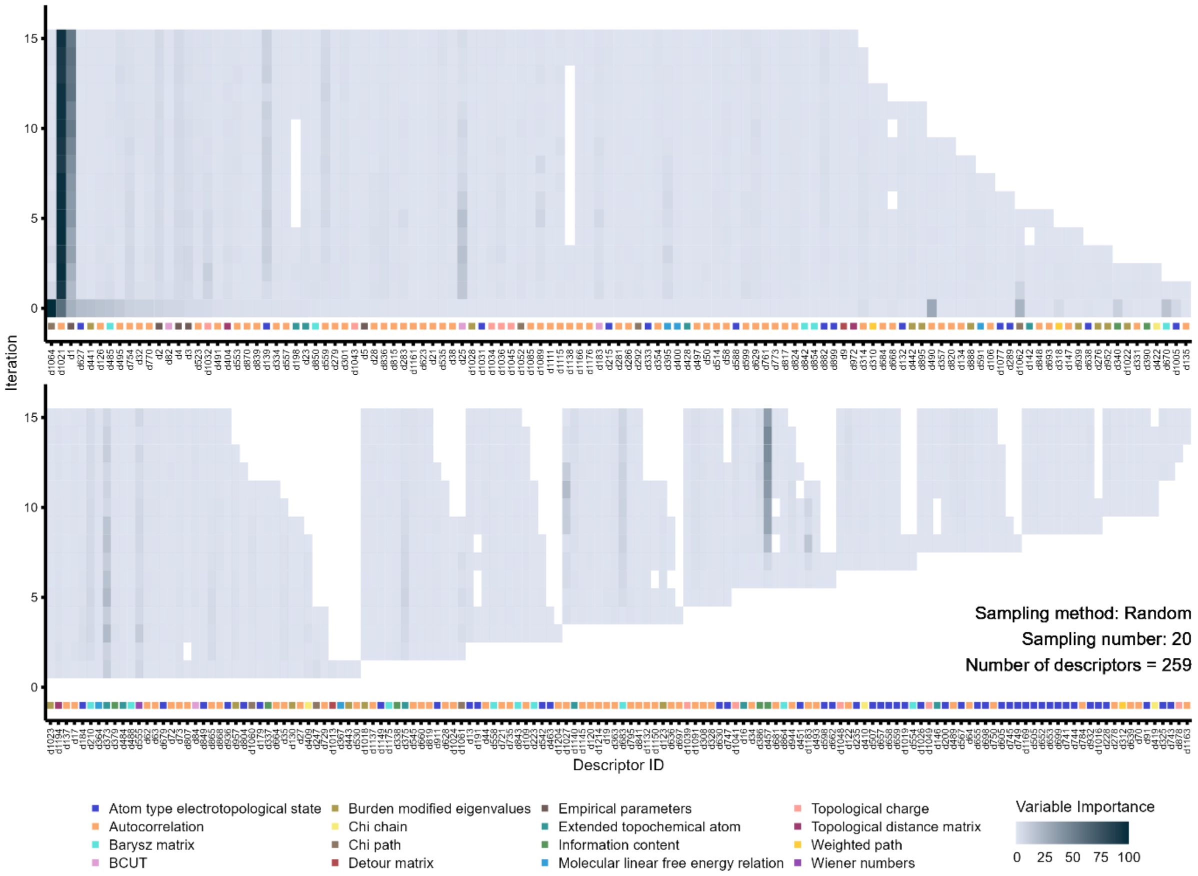

Figure S19. Changes of variable importance in AL iteration for random sampling with n_{sample} = 20.

All descriptors were encoded from d1 to d1225 to simplify the presentation (**Table S5**). The mean variable importance was evaluated overall by 50 repetitions for each iteration after replacing the NA values with zeros. Variances for the original model before AL were shown as the zeroth iteration.

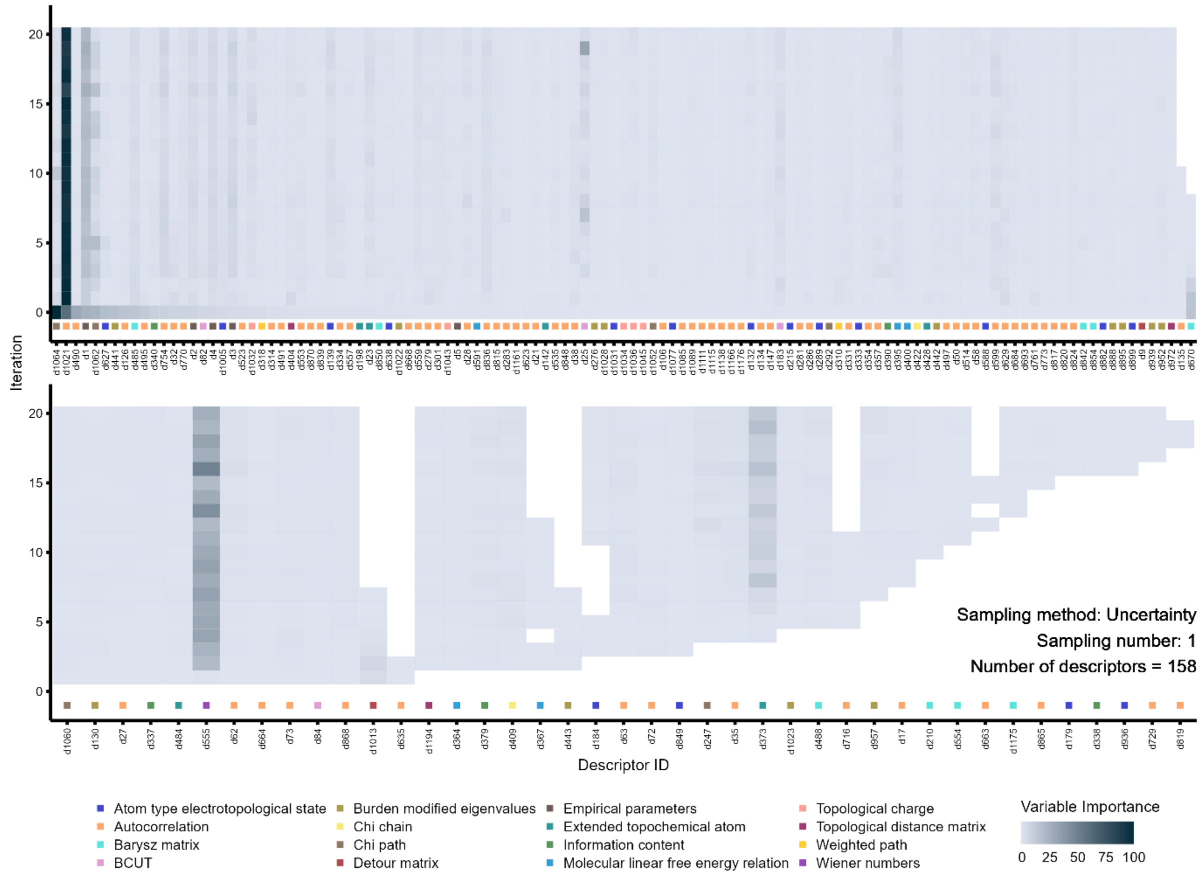

Figure S20. Changes of variable importance in AL iteration for uncertainty-based sampling with n_{sample} = 1.

All descriptors were encoded from d1 to d1225 to simplify the presentation (**Table S5**). The mean variable importance was evaluated overall by 50 repetitions for each iteration after replacing the NA values with zeros. Variances for the original model before AL were shown as the zeroth iteration.

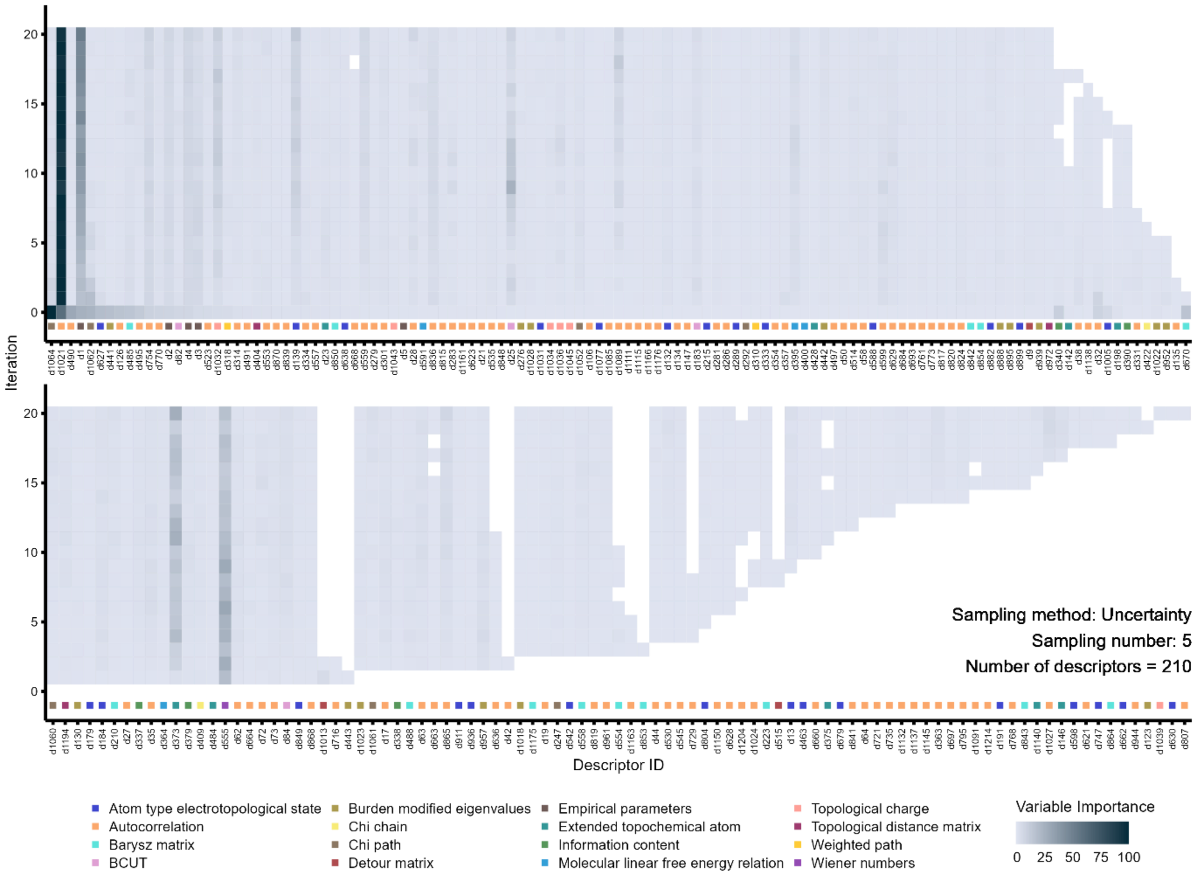

Figure S21. Changes of variable importance in AL iteration for uncertainty-based sampling with n_{sample} = 5.

All descriptors were encoded from d1 to d1225 to simplify the presentation (**Table S5**). The mean variable importance was evaluated overall by 50 repetitions for each iteration after replacing the NA values with zeros. Variances for the original model before AL were shown as the zeroth iteration.

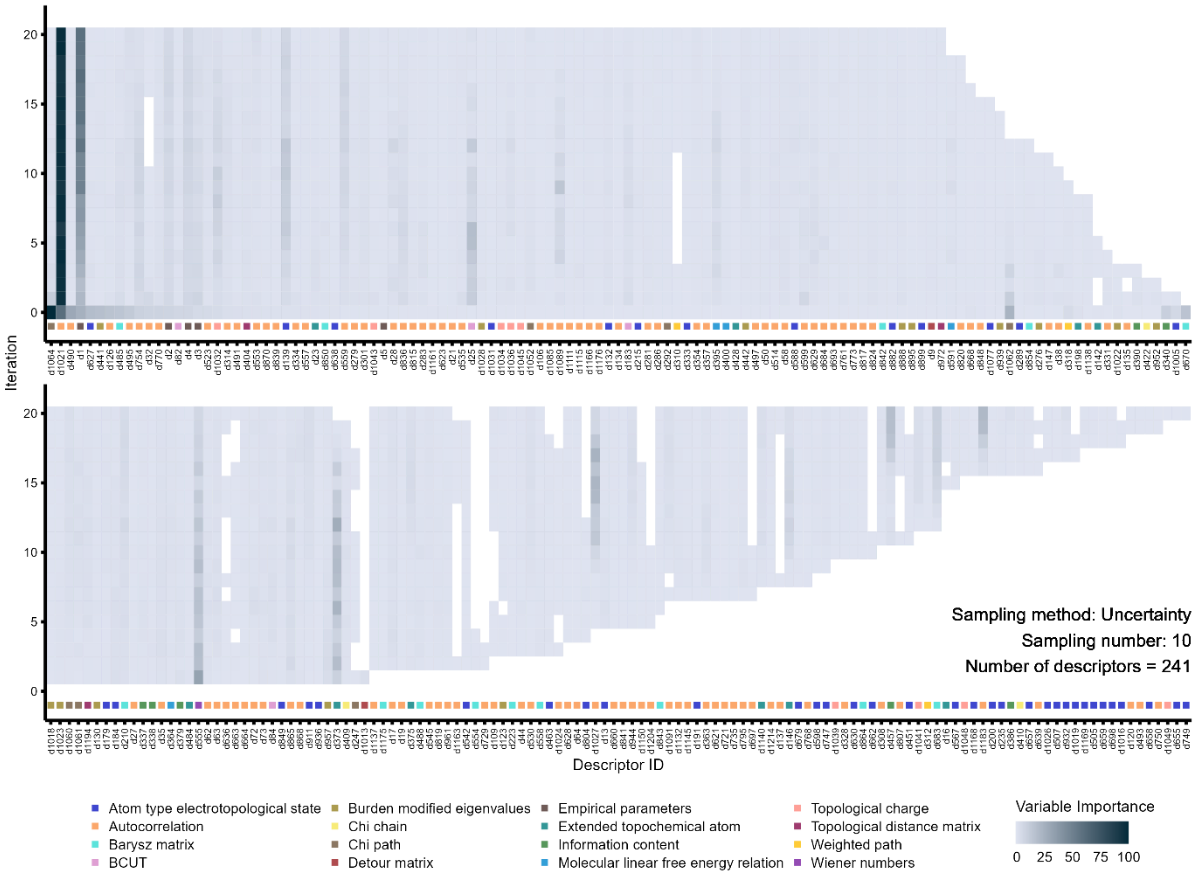

Figure S22. Changes of variable importance in AL iteration for uncertainty-based sampling with n_{sample} = 10.

All descriptors were encoded from d1 to d1225 to simplify the presentation (**Table S5**). The mean variable importance was evaluated overall by 50 repetitions for each iteration after replacing the NA values with zeros. Variances for the original model before AL were shown as the zeroth iteration.

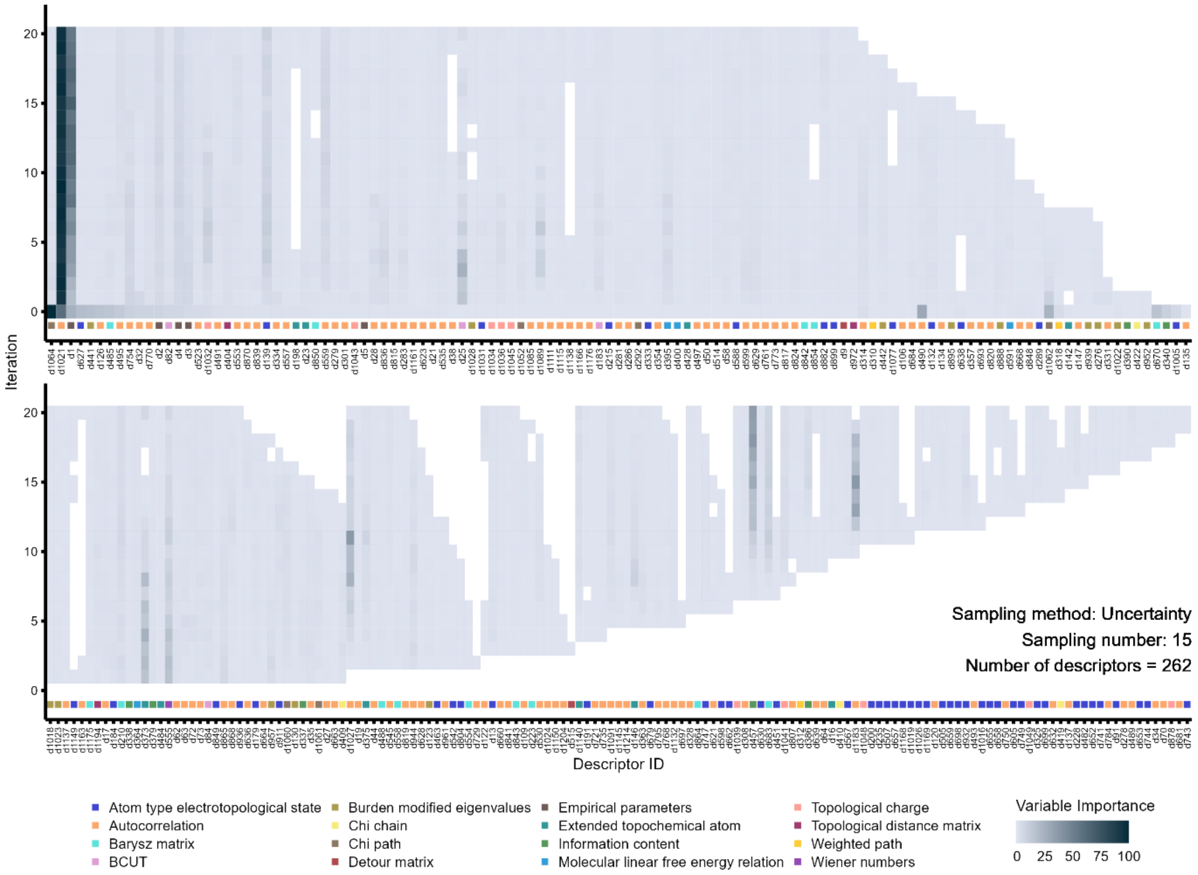

Figure S23. Changes of variable importance in AL iteration for uncertainty-based sampling with n_{sample} = 15.

All descriptors were encoded from d1 to d1225 to simplify the presentation (**Table S5**). The mean variable importance was evaluated overall by 50 repetitions for each iteration after replacing the NA values with zeros. Variances for the original model before AL were shown as the zeroth iteration.

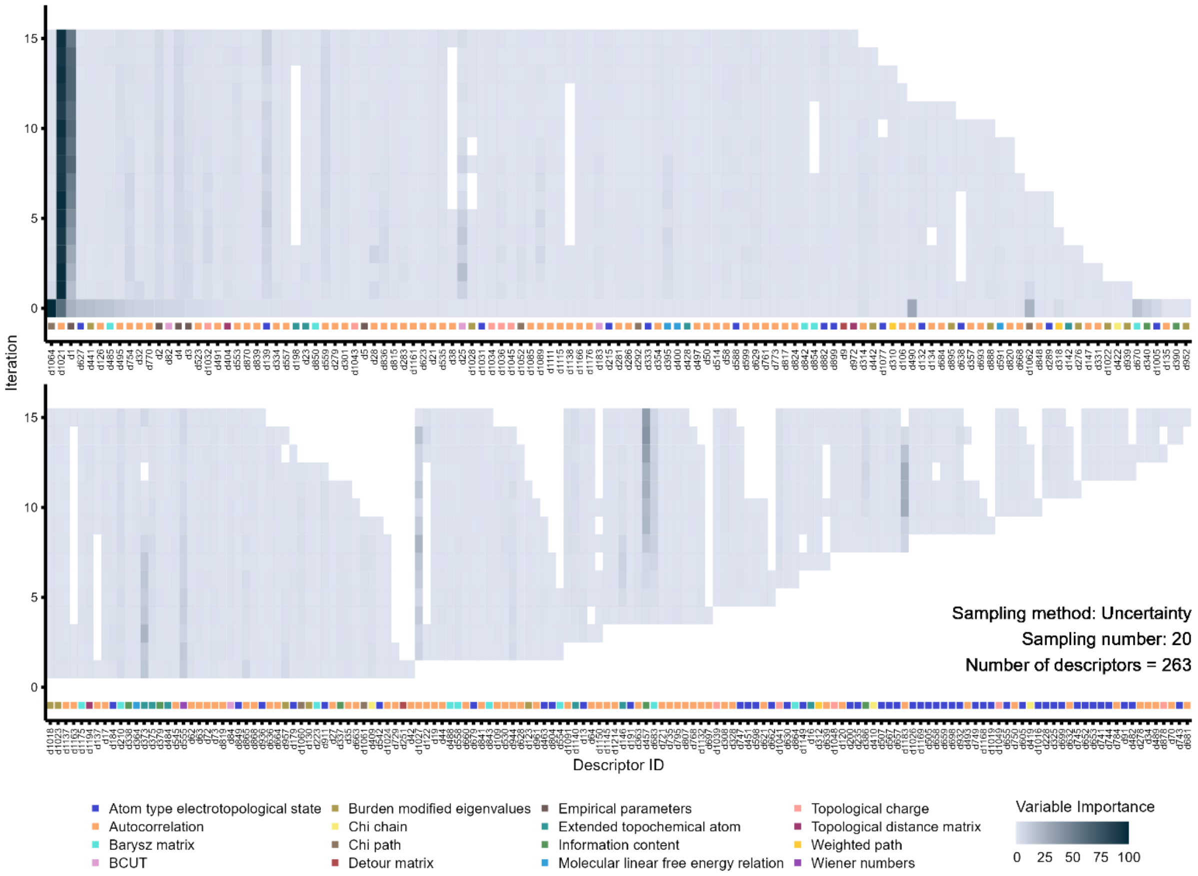

Figure S24. Changes of variable importance in AL iteration for uncertainty-based sampling with n_{sample} = 20.

All descriptors were encoded from d1 to d1225 to simplify the presentation (**Table S5**). The mean variable importance was evaluated overall by 50 repetitions for each iteration after replacing the NA values with zeros. Variances for the original model before AL were shown as the zeroth iteration.

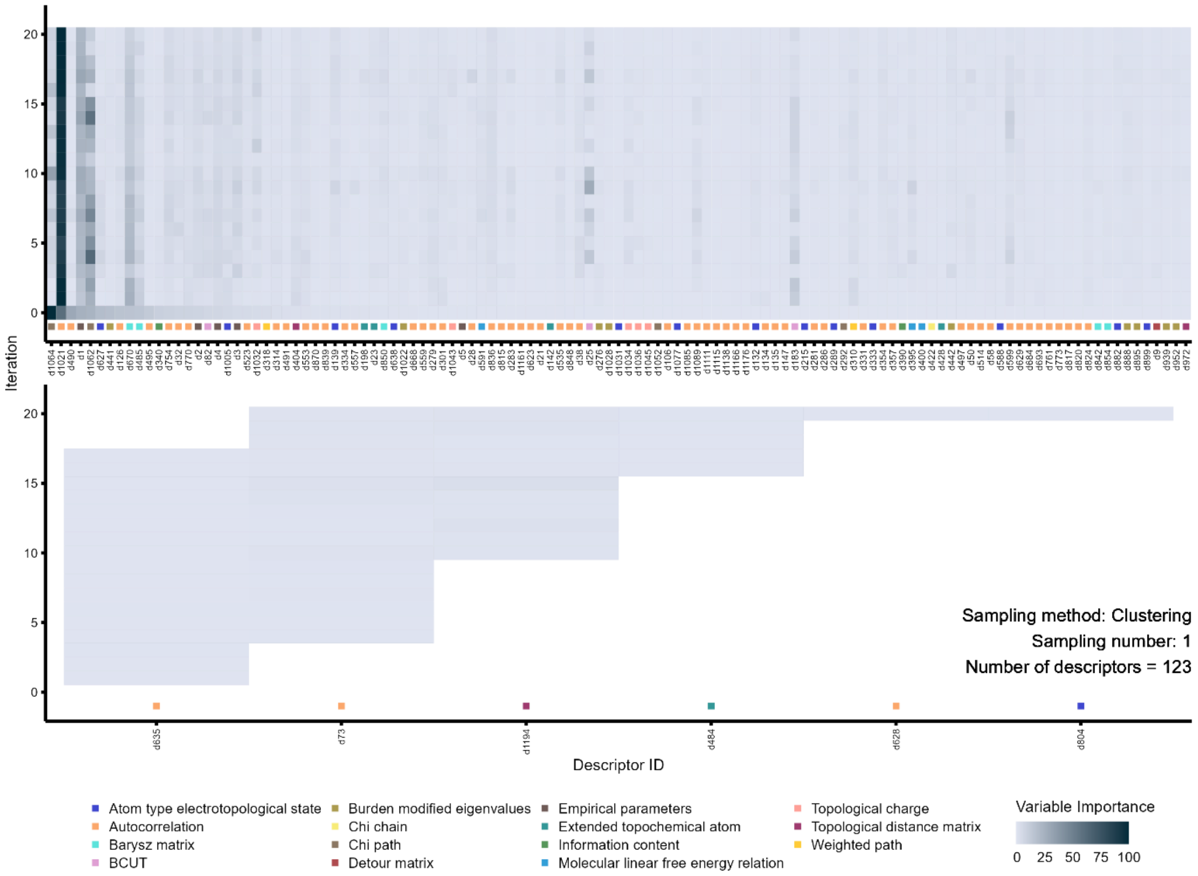

Figure S25. Changes of variable importance in AL iteration for clustering-based sampling with $n_{sample} = 1$.

All descriptors were encoded from d1 to d1225 to simplify the presentation (**Table S5**). The mean variable importance was evaluated overall by 50 repetitions for each iteration after replacing the NA values with zeros. Variances for the original model before AL were shown as the zeroth iteration.

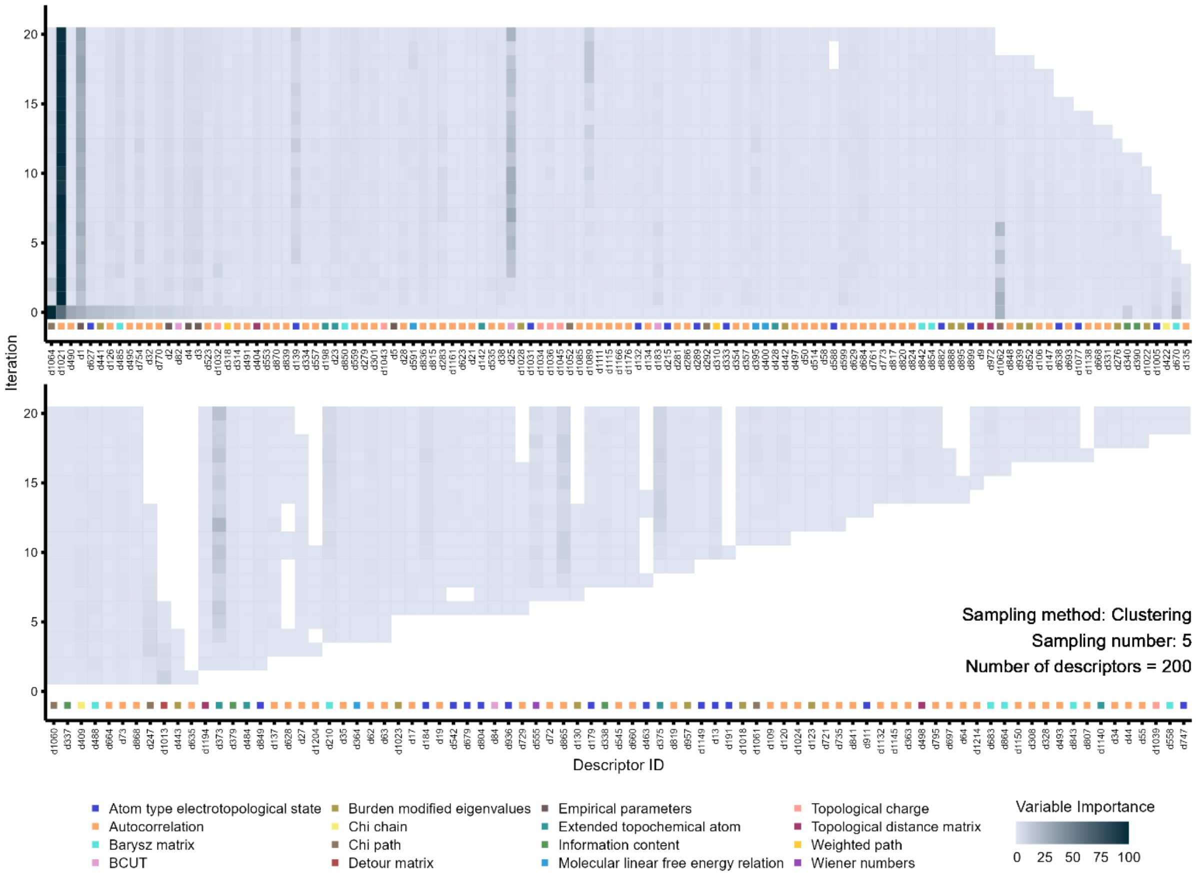

Figure S26. Changes of variable importance in AL iteration for clustering-based sampling with $n_{sample} = 5$.

All descriptors were encoded from d1 to d1225 to simplify the presentation (**Table S5**). The mean variable importance was evaluated overall by 50 repetitions for each iteration after replacing the NA values with zeros. Variances for the original model before AL were shown as the zeroth iteration.

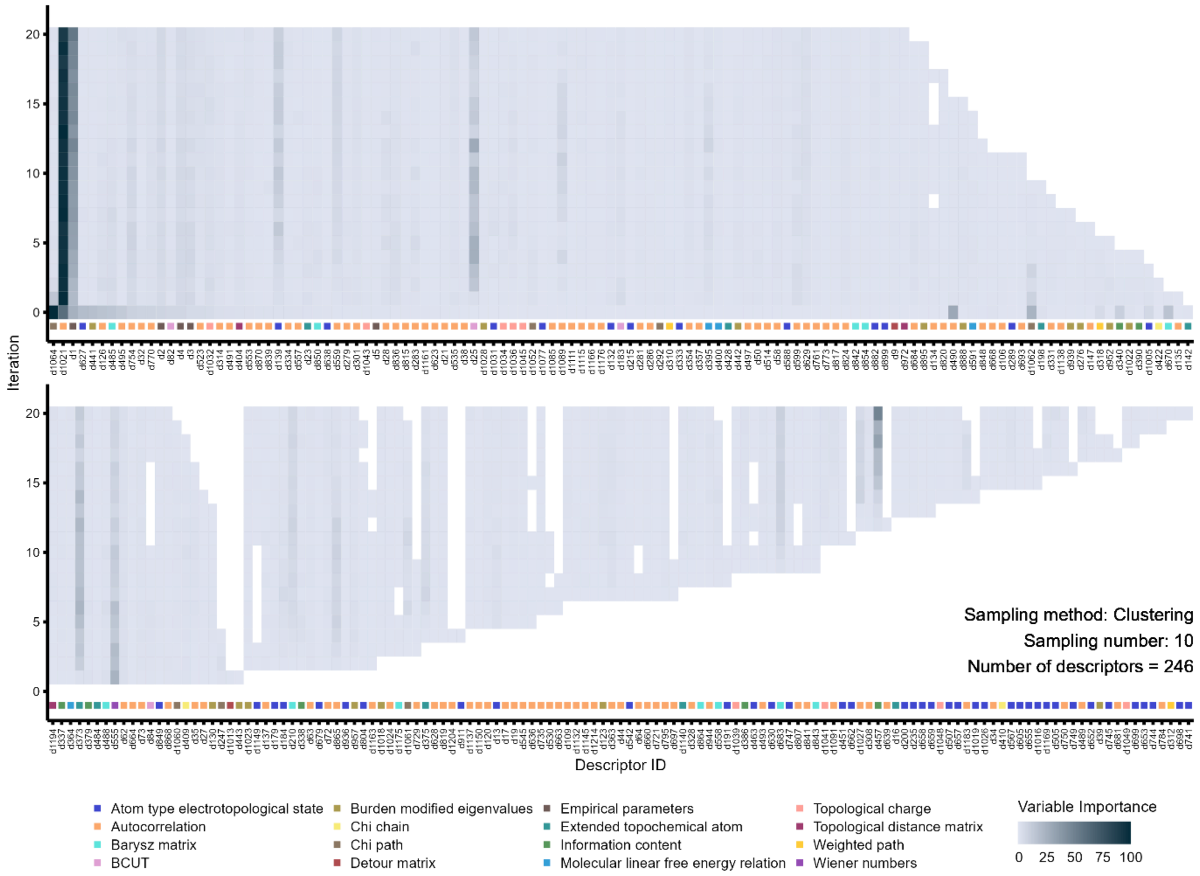

Figure S27. Changes of variable importance in AL iteration for clustering-based sampling with $n_{sample} = 10$.

All descriptors were encoded from d1 to d1225 to simplify the presentation (**Table S5**). The mean variable importance was evaluated overall by 50 repetitions for each iteration after replacing the NA values with zeros. Variances for the original model before AL were shown as the zeroth iteration.

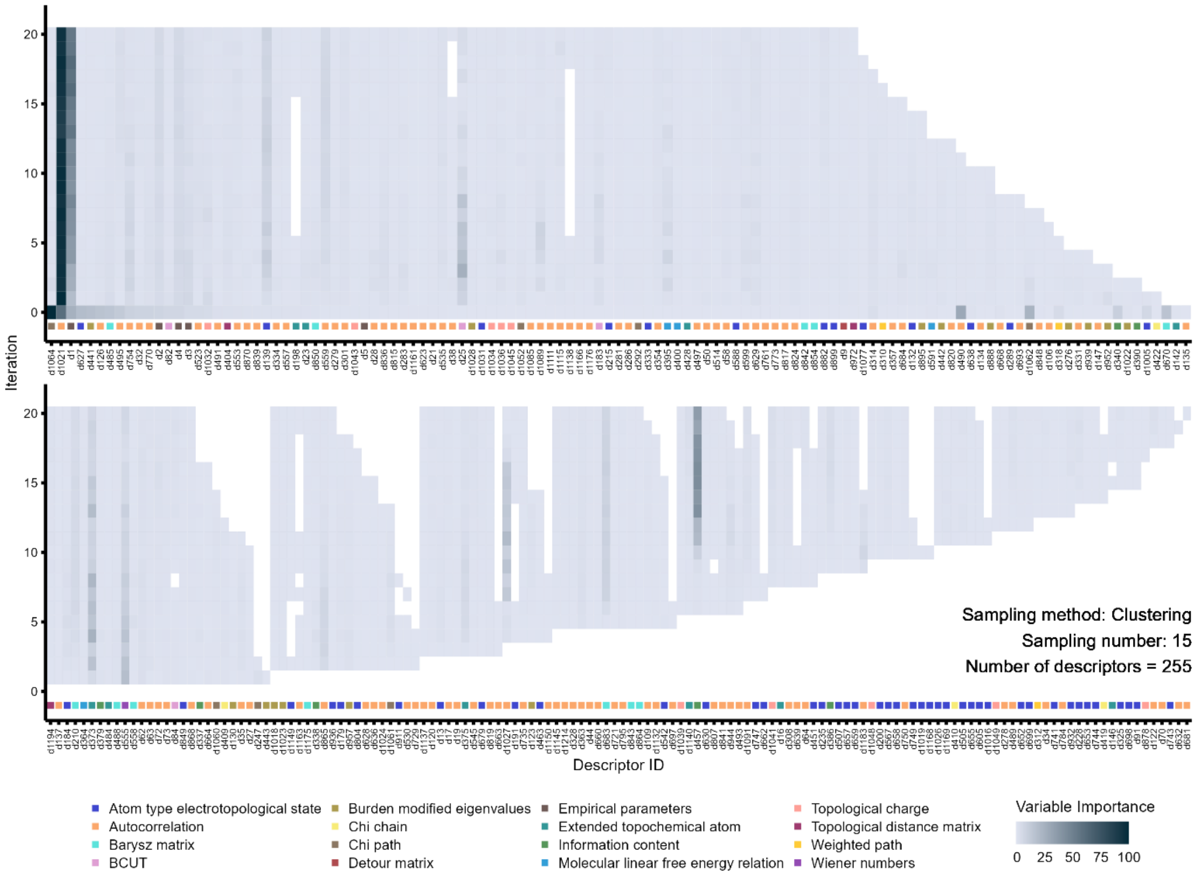

Figure S28. Changes of variable importance in AL iteration for clustering-based sampling with n_{sample} = 15.

All descriptors were encoded from d1 to d1225 to simplify the presentation (**Table S5**). The mean variable importance was evaluated overall by 50 repetitions for each iteration after replacing the NA values with zeros. Variances for the original model before AL were shown as the zeroth iteration.

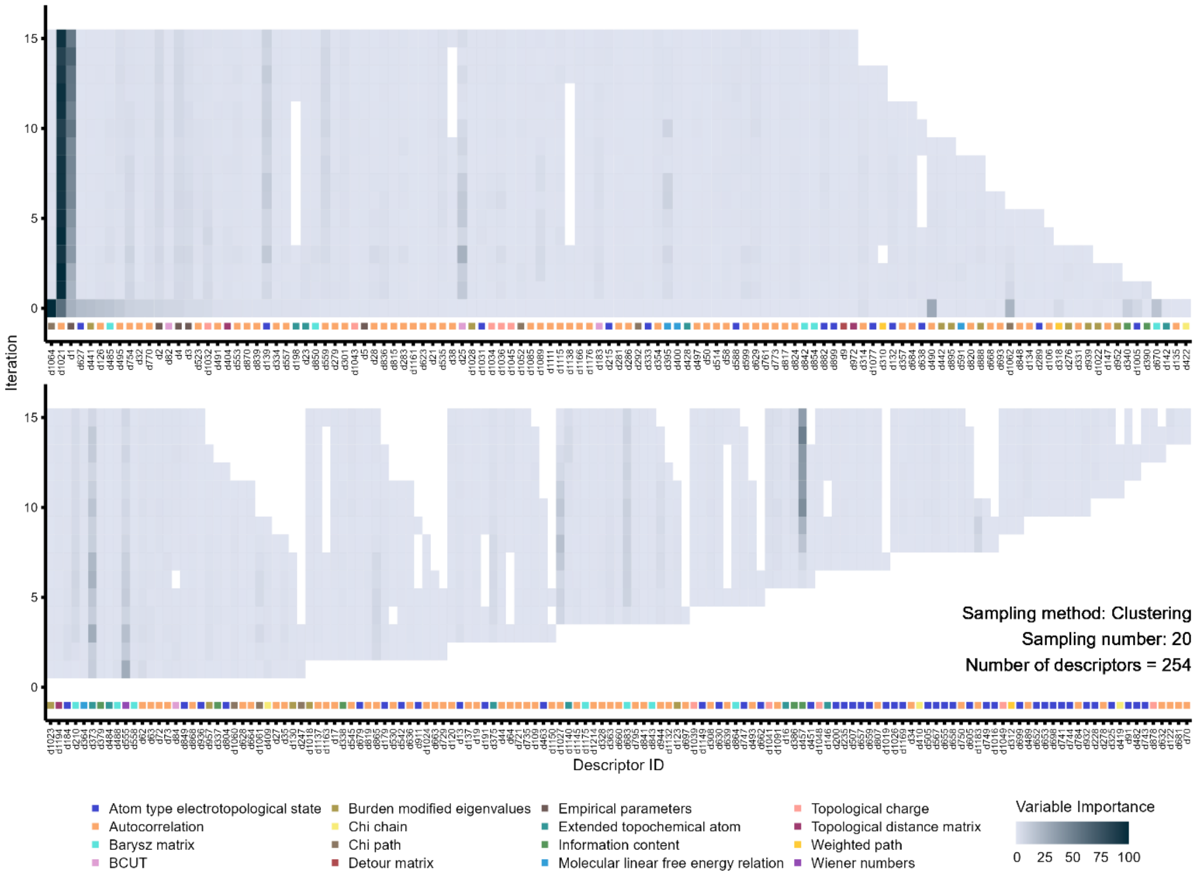

Figure S29. Changes of variable importance in AL iteration for clustering-based sampling with n_{sample} = 20.

All descriptors were encoded from d1 to d1225 to simplify the presentation (**Table S5**). The mean variable importance was evaluated overall by 50 repetitions for each iteration after replacing the NA values with zeros. Variances for the original model before AL were shown as the zeroth iteration.

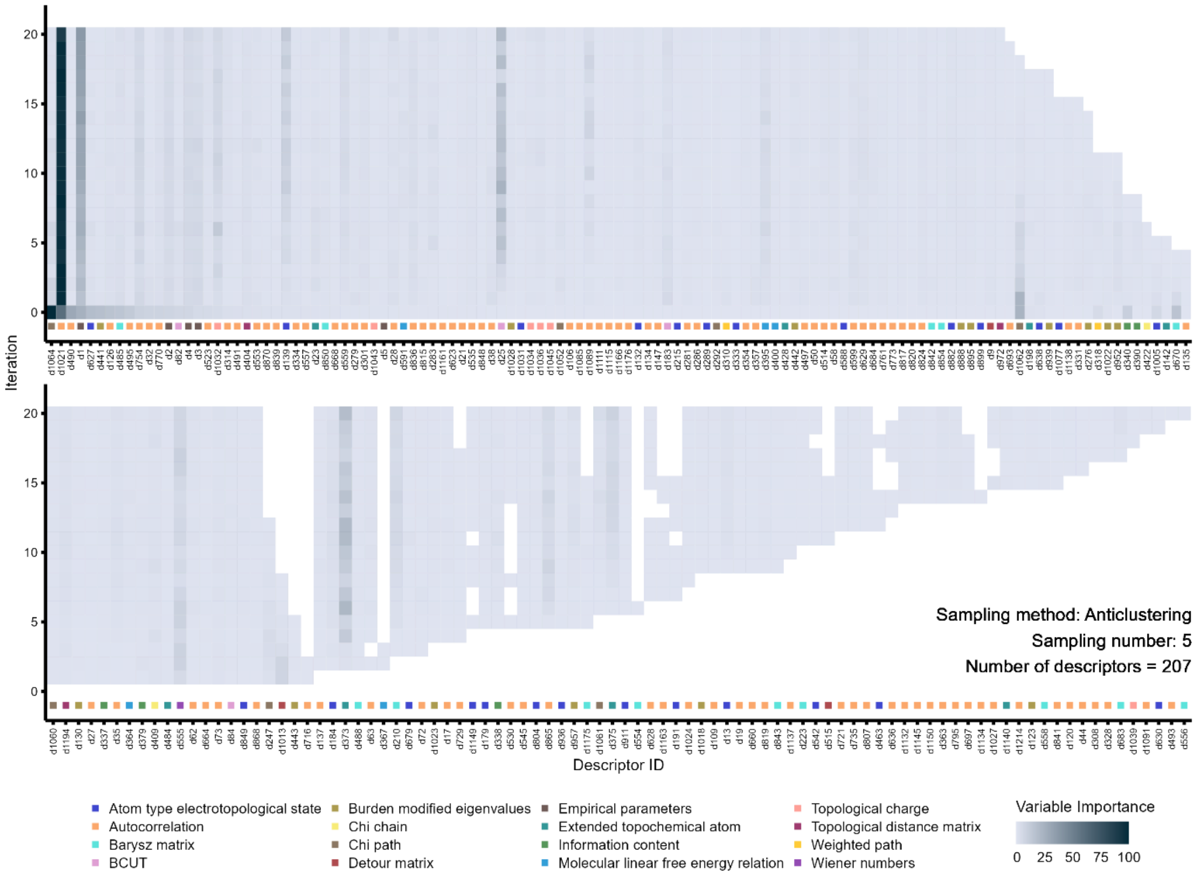

Figure S30. Changes of variable importance in AL iteration for anti-clustering-based sampling with n_{sample} = 5.

All descriptors were encoded from d1 to d1225 to simplify the presentation (**Table S5**). The mean variable importance was evaluated overall by 50 repetitions for each iteration after replacing the NA values with zeros. Variances for the original model before AL were shown as the zeroth iteration.

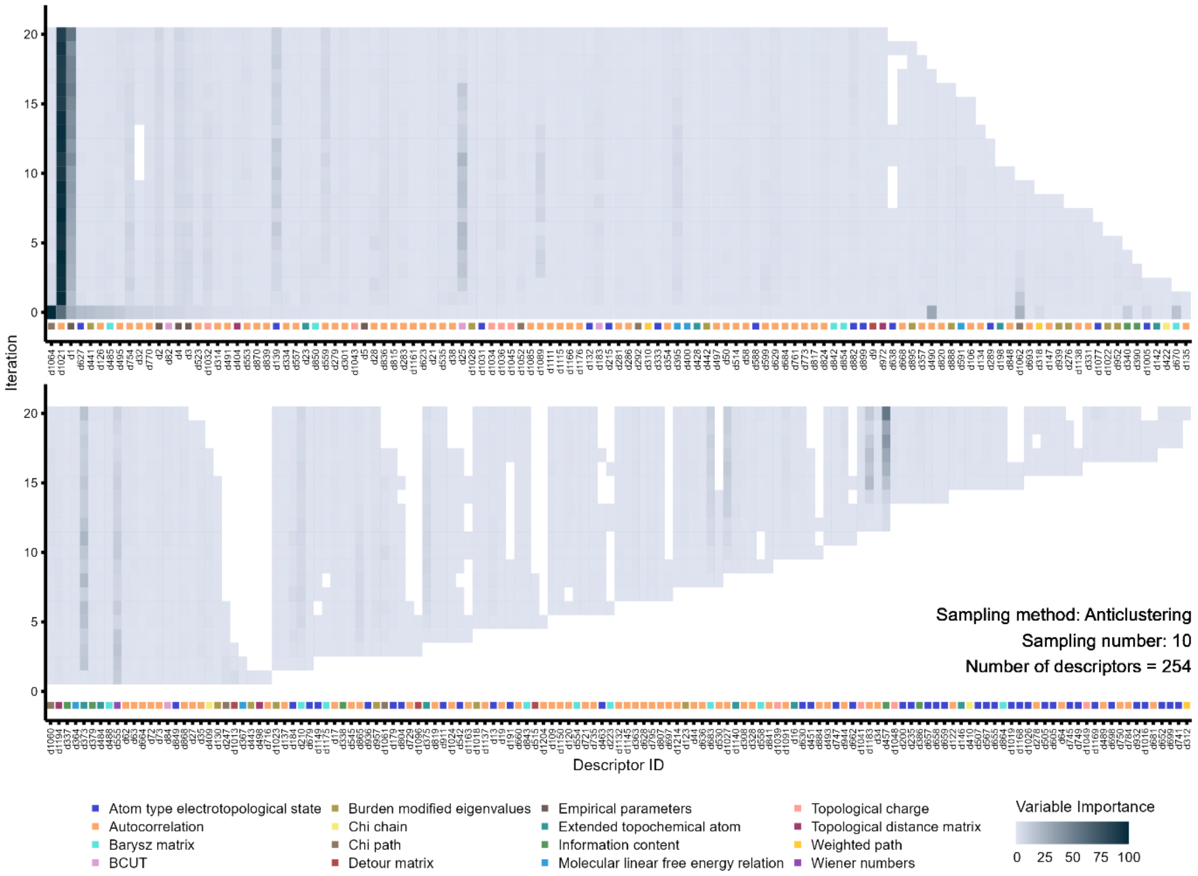

Figure S31. Changes of variable importance in AL iteration for anti-clustering-based sampling with n_{sample} = 10.

All descriptors were encoded from d1 to d1225 to simplify the presentation (**Table S5**). The mean variable importance was evaluated overall by 50 repetitions for each iteration after replacing the NA values with zeros. Variances for the original model before AL were shown as the zeroth iteration.

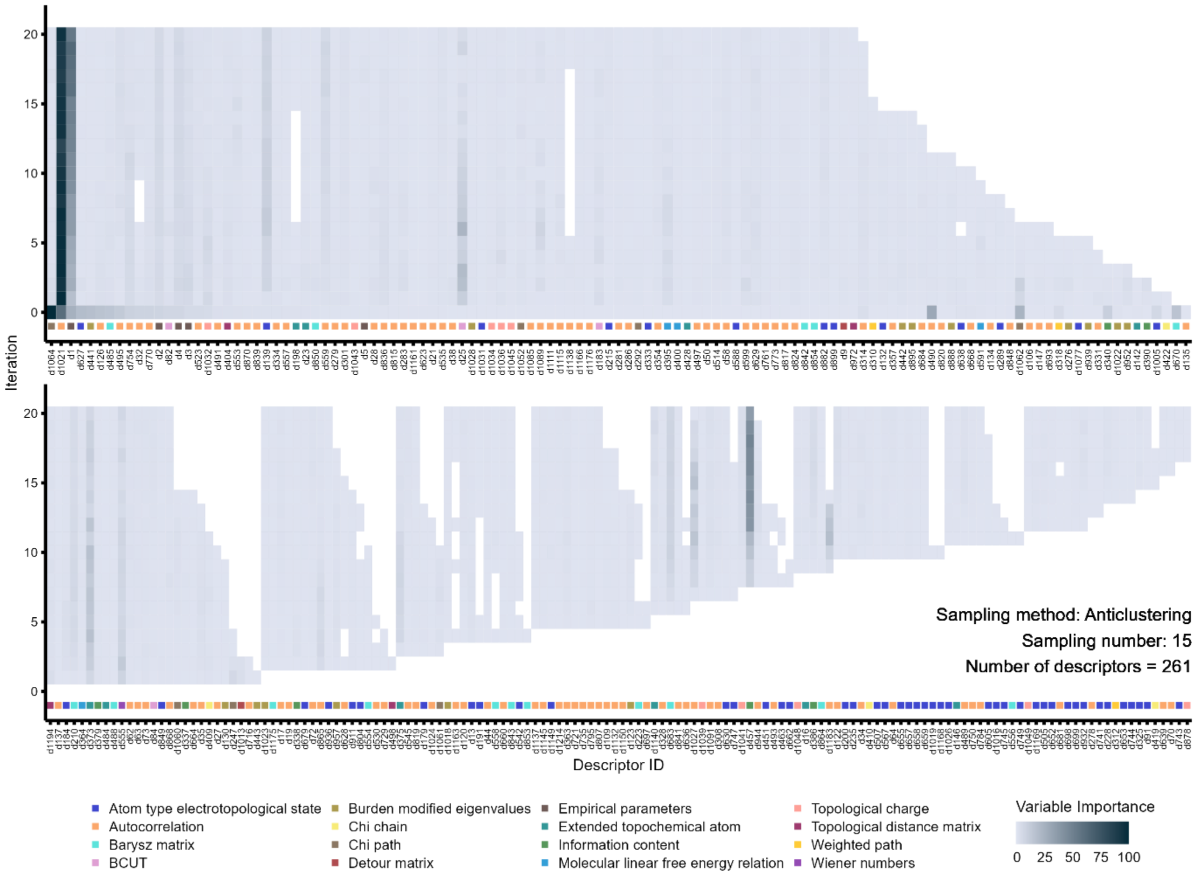

Figure S32. Changes of variable importance in AL iteration for anti-clustering-based sampling with n_{sample} = 15.

All descriptors were encoded from d1 to d1225 to simplify the presentation (**Table S5**). The mean variable importance was evaluated overall by 50 repetitions for each iteration after replacing the NA values with zeros. Variances for the original model before AL were shown as the zeroth iteration.

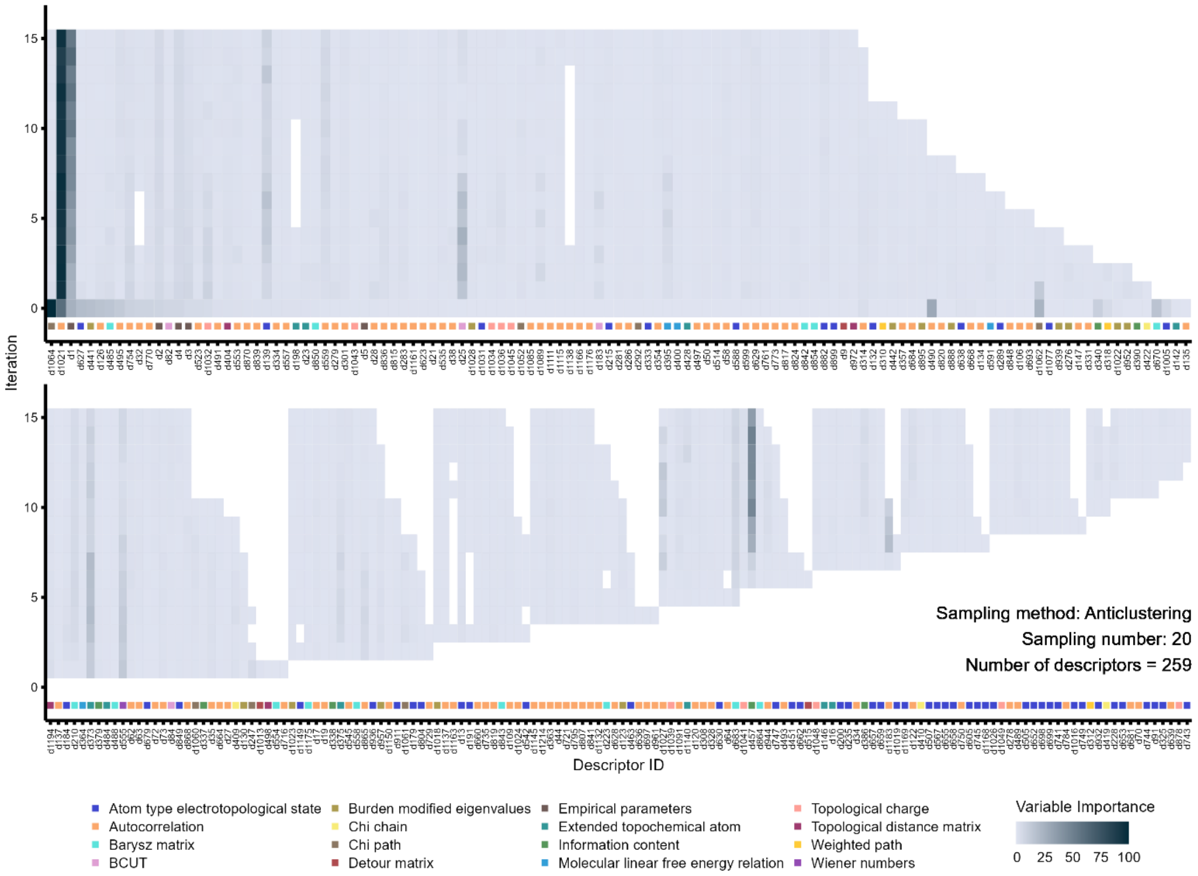

Figure S33. Changes of variable importance in AL iteration for anti-clustering-based sampling with n_{sample} = 20.

All descriptors were encoded from d1 to d1225 to simplify the presentation (**Table S5**). The mean variable importance was evaluated overall by 50 repetitions for each iteration after replacing the NA values with zeros. Variances for the original model before AL were shown as the zeroth iteration.

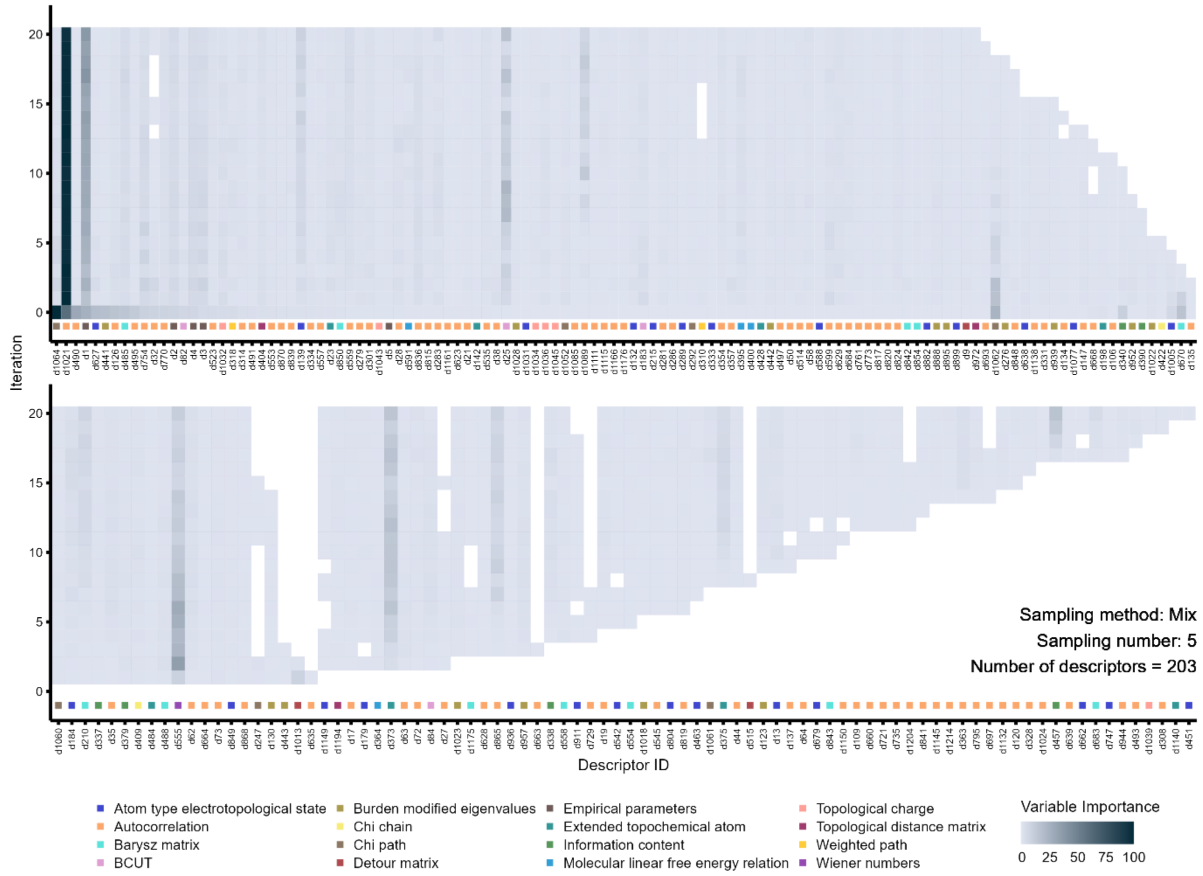

Figure S34. Changes of variable importance in AL iteration for mix sampling with n_{sample} = 5.

All descriptors were encoded from d1 to d1225 to simplify the presentation (**Table S5**). The mean variable importance was evaluated overall by 50 repetitions for each iteration after replacing the NA values with zeros. Variances for the original model before AL were shown as the zeroth iteration.

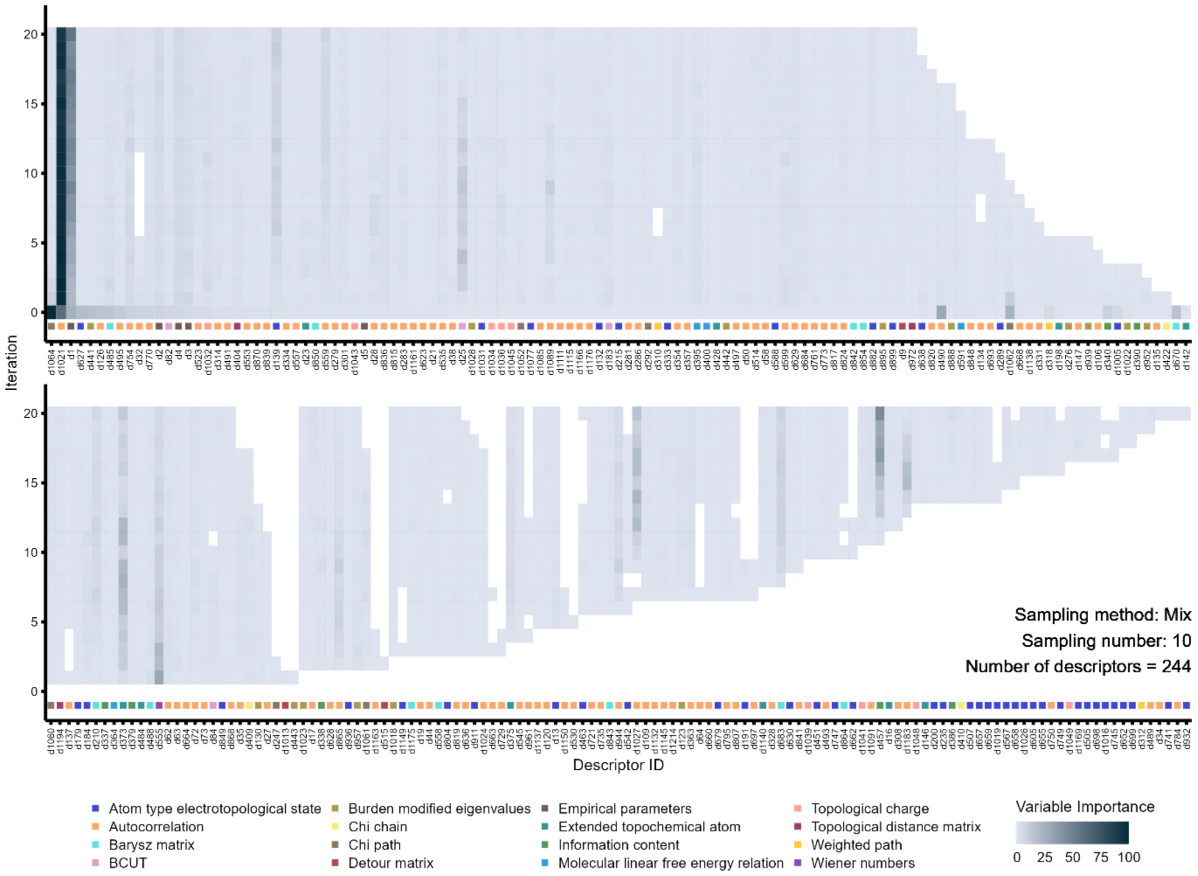

Figure S35. Changes of variable importance in AL iteration for mix sampling with n_{sample} = 10.

All descriptors were encoded from d1 to d1225 to simplify the presentation (**Table S5**). The mean variable importance was evaluated overall by 50 repetitions for each iteration after replacing the NA values with zeros. Variances for the original model before AL were shown as the zeroth iteration.

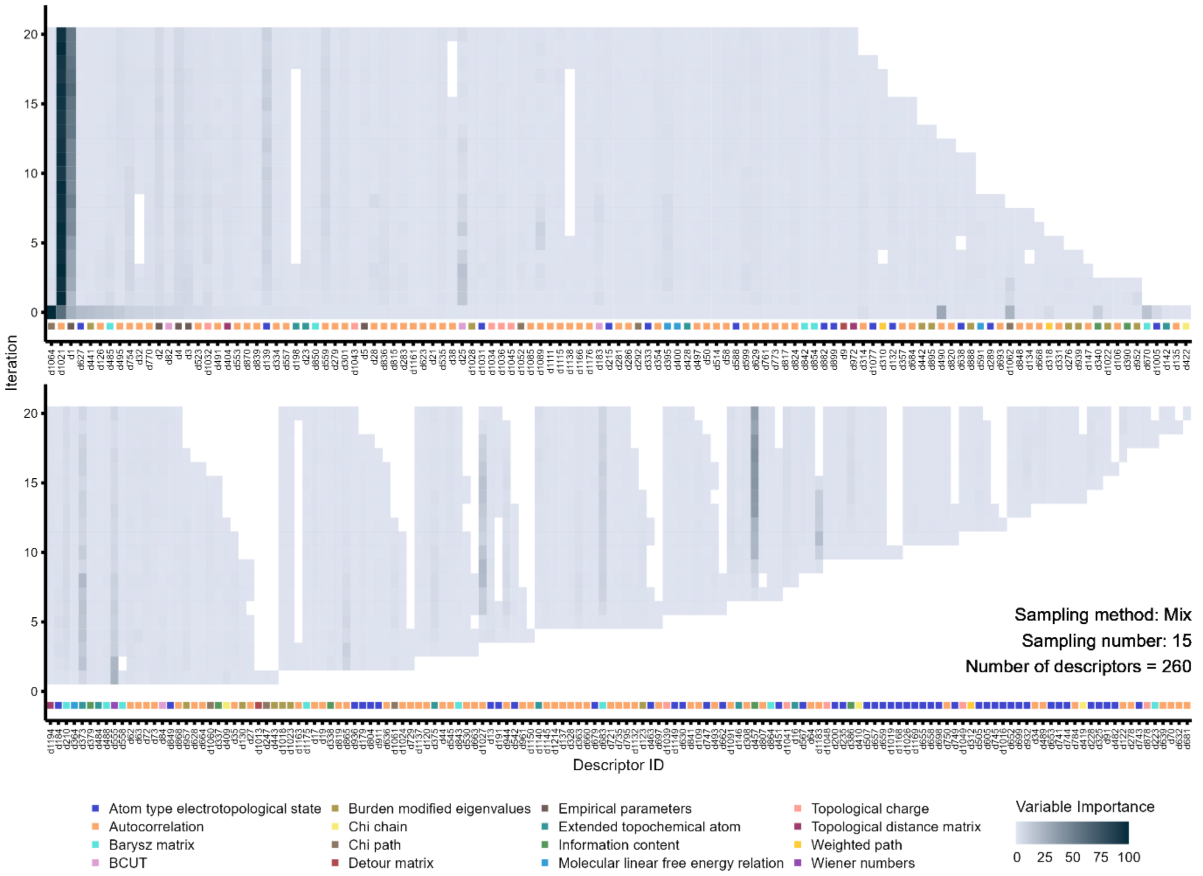

Figure S36. Changes of variable importance in AL iteration for mix sampling with n_{sample} = 15.

All descriptors were encoded from d1 to d1225 to simplify the presentation (**Table S5**). The mean variable importance was evaluated overall by 50 repetitions for each iteration after replacing the NA values with zeros. Variances for the original model before AL were shown as the zeroth iteration.

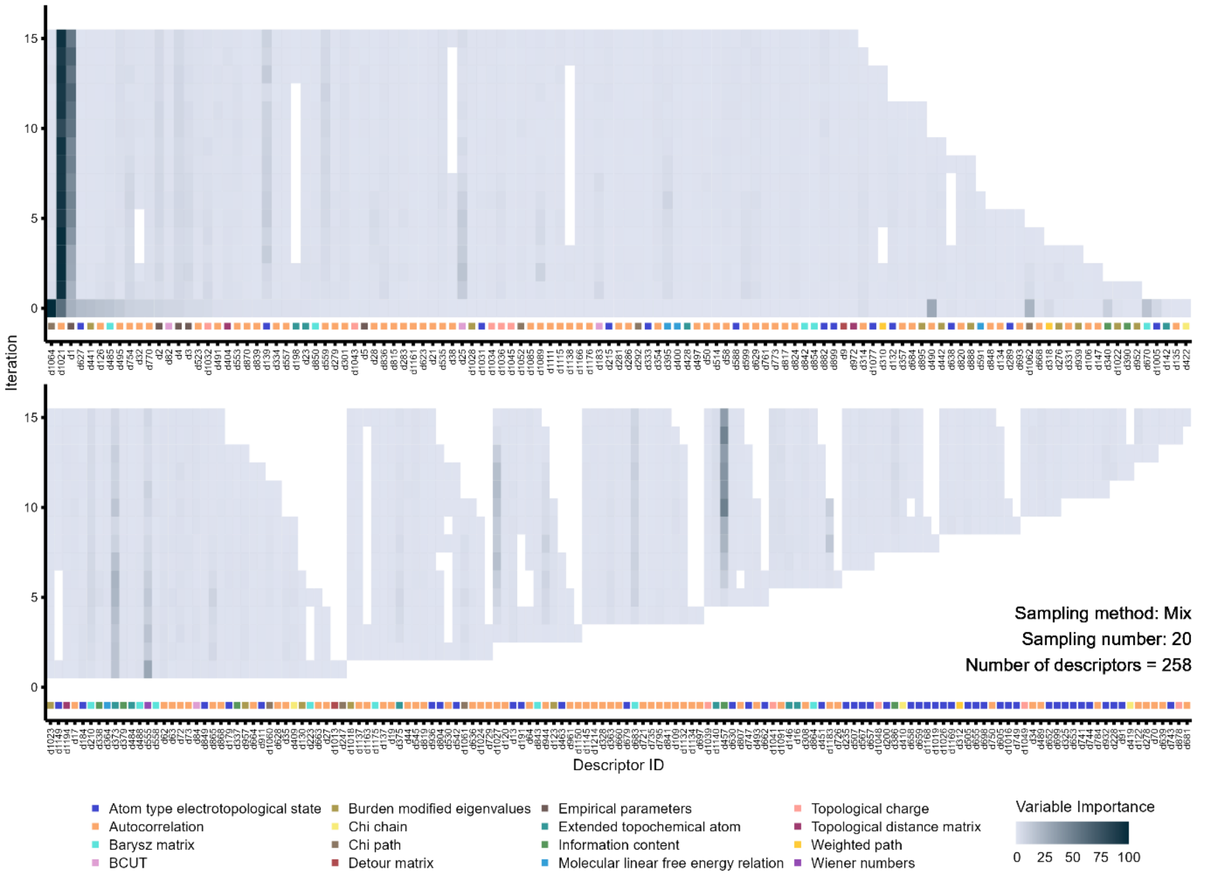

Figure S37. Changes of variable importance in AL iteration for mix sampling with n_{sample} = 20.

All descriptors were encoded from d1 to d1225 to simplify the presentation (**Table S5**). The mean variable importance was evaluated overall by 50 repetitions for each iteration after replacing the NA values with zeros. Variances for the original model before AL were shown as the zeroth iteration.

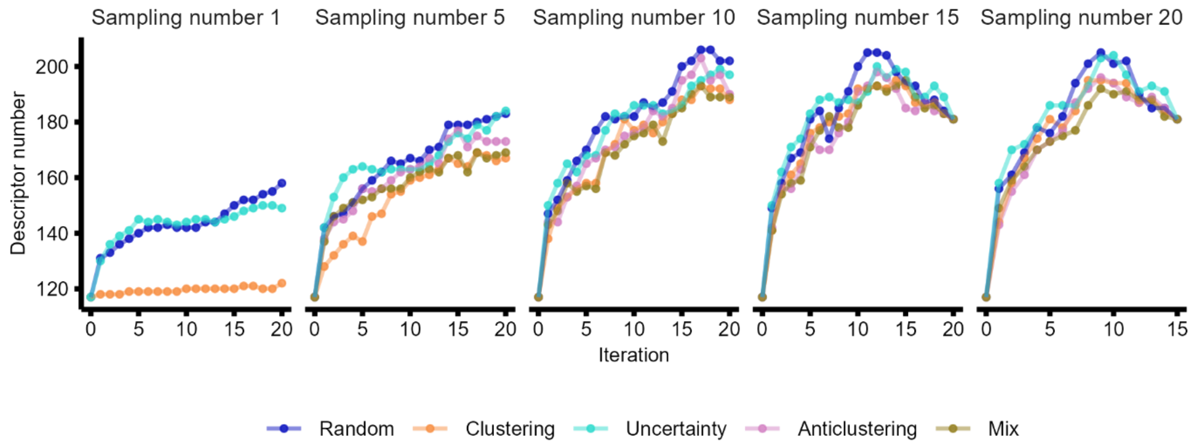

Figure S38. The number of descriptors in each iteration for various algorithms

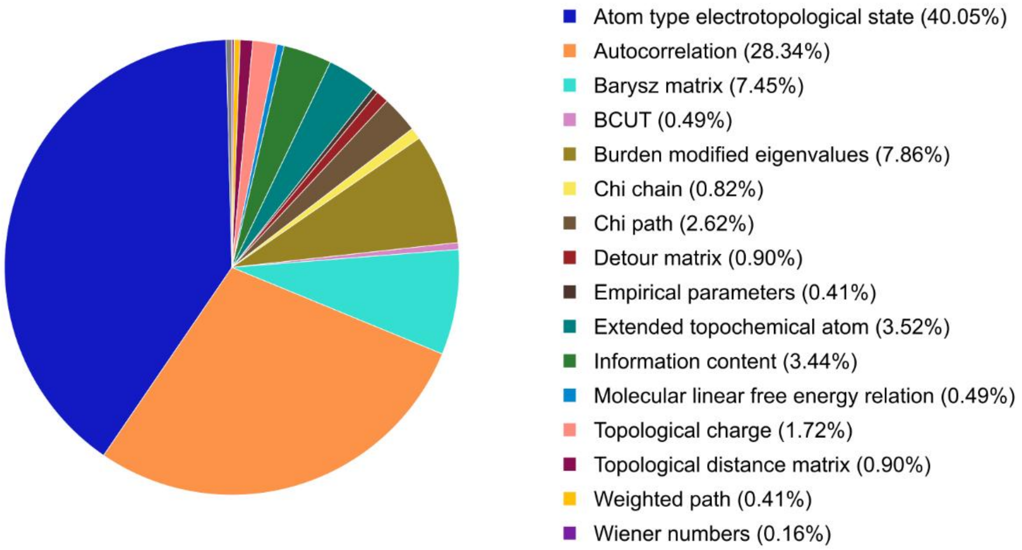

Figure S39. Proportions of the descriptor types for PaDEL descriptors[2]

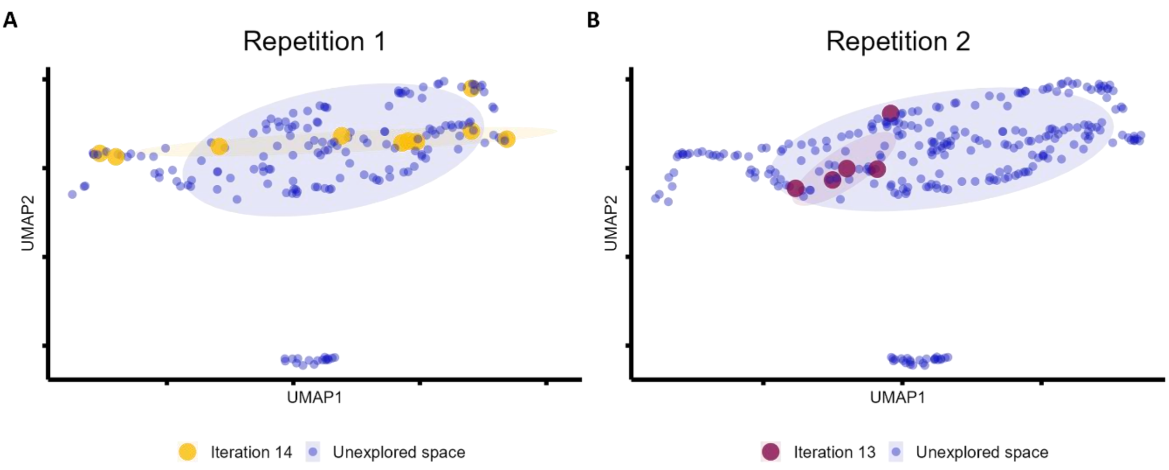

Figure S40. Demonstration cases of suggestions from random approach with insufficient diversity. (A) n_{sample} = 10, the 14th iteration, and the 1st repetition. (B) n_{sample} = 5, the 13th iteration, and the 2nd repetition.

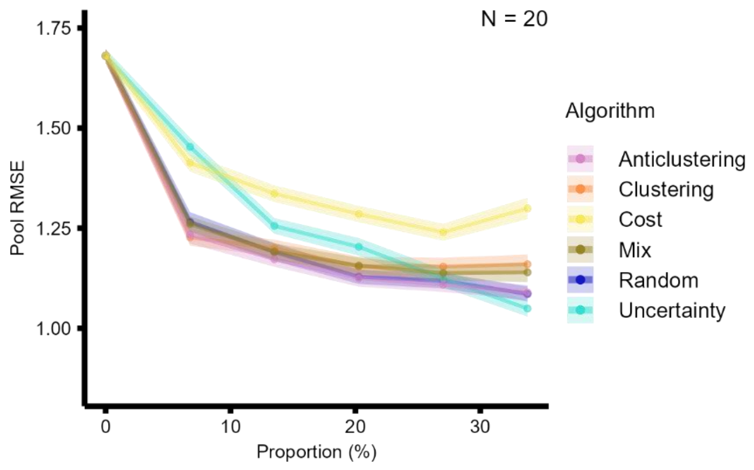

Figure S41. AL performance evaluation with selected algorithms compared to cost-based sampling

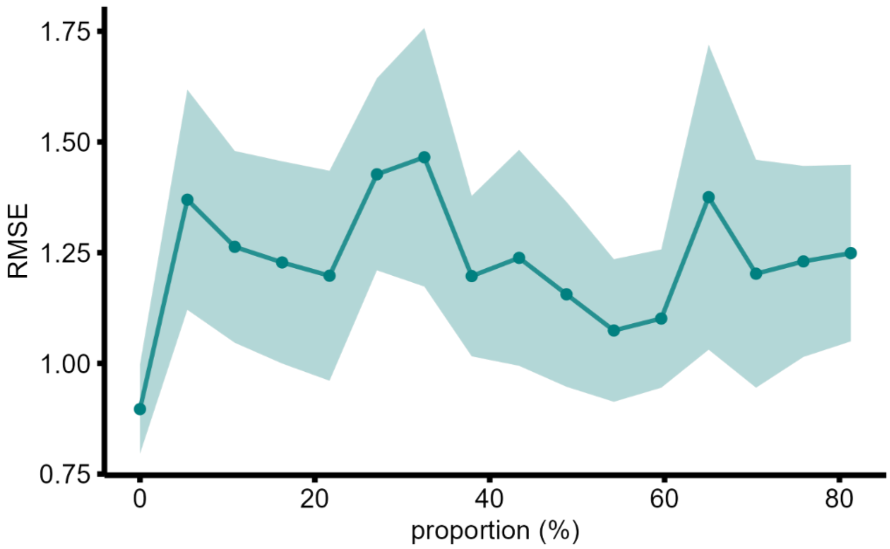

Figure S42. Performance of the ML log*IE* prediction models on 20% of the explored space with random sampling for 15 repetitions. The x-axis was the proportion of the labeled compounds over the compounds in the whole unexplored space with each iteration step. Each point in the plots represented the average result from all repetitions, and the color ranges were inferred from the SEM.

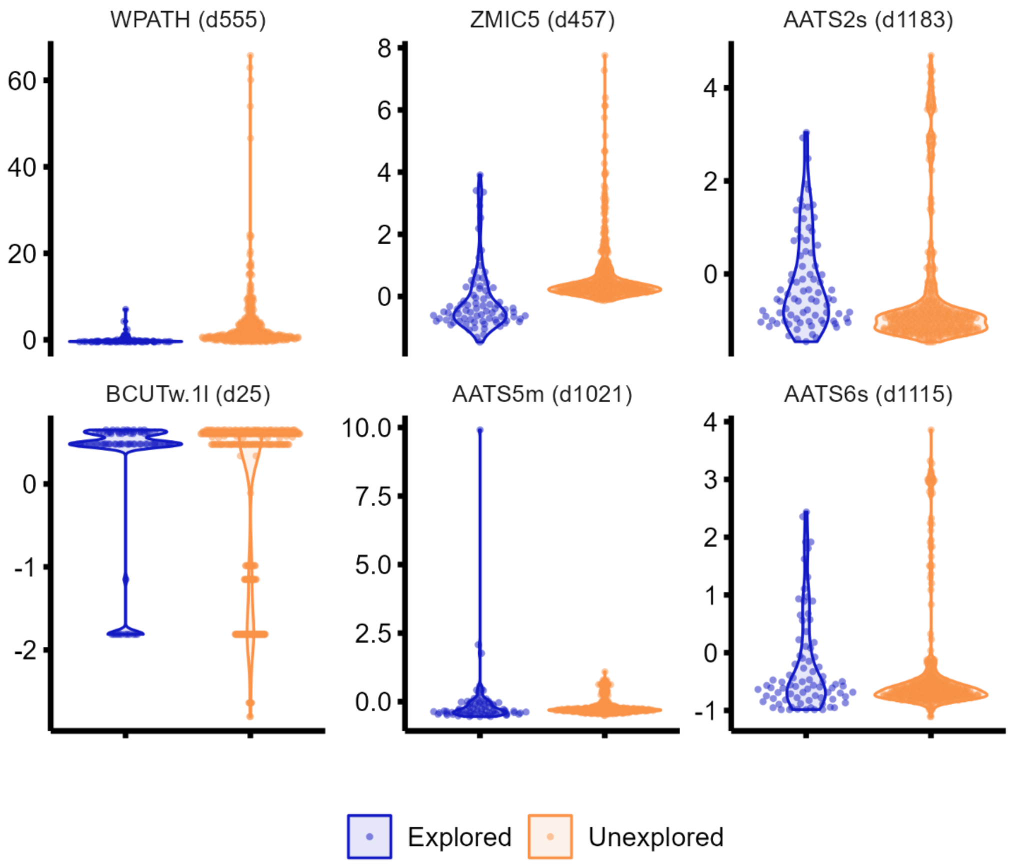

Figure S43. Distribution comparison between the explored space and unexplored space for descriptors in interest

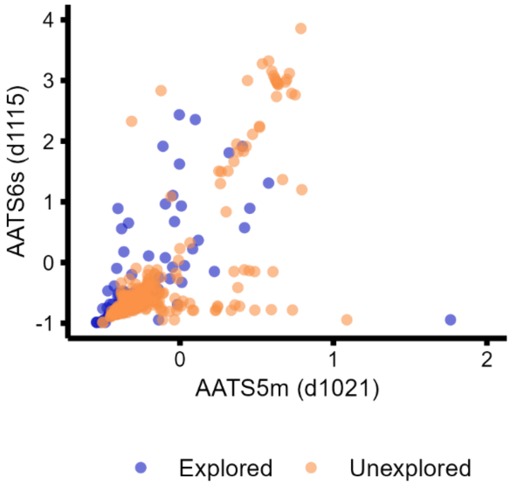

Figure S44. Correlation between the explored space and unexplored space for descriptors AATS5m (d1021) and AATS6s (d1115)

Reference

(1) Pluskal, T.; Castillo, S.; Villar-Briones, A.; Orešič, M. MZmine 2: Modular Framework for Processing, Visualizing, and Analyzing Mass Spectrometry-Based Molecular Profile Data. *BMC Bioinformatics* **2010**, *11* (1), 395. https://doi.org/10.1186/1471-2105-11-395.

(2) Yap, C. W. PaDEL-Descriptor: An Open Source Software to Calculate Molecular Descriptors and Fingerprints. *J Comput Chem* **2011**, *32* (7), 1466–1474. https://doi.org/10.1002/jcc.21707.

(3) Djoumbou Feunang, Y.; Eisner, R.; Knox, C.; Chepelev, L.; Hastings, J.; Owen, G.; Fahy, E.; Steinbeck, C.; Subramanian, S.; Bolton, E.; Greiner, R.; Wishart, D. S. ClassyFire: Automated Chemical Classification with a Comprehensive, Computable Taxonomy. *J. Cheminformatics* **2016**, *8* (1), 61. https://doi.org/10.1186/s13321-016-0174-y.
